# Optimisation of ultrasound-assisted deep eutectic solvent extraction of carbohydrates and proteins from farmed brown seaweed (*Saccharina latissima*) using response surface methodology

**DOI:** 10.1016/j.ultsonch.2026.108144

**Published:** 2026-10-30

**Authors:** Rifna E. Jerome, Nilushni Sivapragasam, Brijesh K. Tiwari, Gaurav Rajauria

**Affiliations:** a SUSFERM Centre for Sustainable Fermentation and Bioprocessing Systems for Food and the Bioeconomy, University College Cork, Cork T12 YN60, Ireland; b School of Microbiology, University College Cork, Cork T12 YN60, Ireland; c School of Food and Nutritional Sciences, University College Cork, Cork T12 YN60, Ireland; d Department of Food Chemistry and Technology, Teagasc Food Research Centre, Ashtown, Dublin D15 KN3K, Ireland

**Keywords:** Ultrasound-assisted extraction, Seaweed, Deep eutectic solvents, Box-Behnken design, Proteins, Total soluble carbohydrates, Antioxidant capacity, Process intensification

## Abstract

Seaweeds are emerging as versatile and promising sources of bioactive compounds with applications in food, feed, and nutraceuticals. This study explored ultrasound-assisted extraction using a deep eutectic solvent (choline chloride: lactic acid) to enhance the co-extraction of proteins and soluble carbohydrates from *Saccharina latissima*. Extraction parameters, ultrasound amplitude (70–90%), sonication time (5–15  min), and solid-to-solvent ratio (3–7%) were optimised using response surface methodology with a Box-Behnken design.

Total protein content (TPC) and total soluble carbohydrate content (TSC) were used as the two primary responses for model development and numerical optimisation, while protein recovery yield (PRY) and carbohydrate recovery yield (CRY) were calculated from the corresponding TPC and TSC values. The optimised conditions (88% amplitude, 12 min sonication, and 3% solid-to-solvent ratio) yielded a TPC of 50.98 mg BSAE/g of ethanol-pretreated and dried seaweed and a TSC of 431.09 mg GE/g of ethanol-pretreated and dried seaweed, corresponding to protein and carbohydrate recovery yields of 56.58% and 83.54%, respectively. The optimised condition was validated experimentally. The developed models for the two responses were highly significant (p < 0.0001), with coefficients of determination (R^2^) exceeding 0.98. The optimised ultrasound-assisted deep eutectic solvent (US-DES) extraction approach significantly enhanced antioxidant capacity, achieving 73.46 ± 1.3% DPPH radical scavenging activity and 134.48 ± 1.67  µM TE/mL of extract for FRAP. Fucose content was maximally recovered under the same conditions (20.56 ± 0.46  mg/g of ethanol-pretreated and dried seaweed). FTIR and SEM analyses revealed changes in the biomass functional-group profiles and surface morphology following extraction. Overall, this study demonstrates the potential of ultrasound-assisted DES extraction for recovering protein- and carbohydrate-rich fractions from *S. latissima*, with promising antioxidant capacity.

## Highlights

• US-DES extraction of *S. latissima* was optimised by RSM.
• US-DES improved carbohydrate and protein recovery over individual methods.
• Optimised US-DES achieved 83.54% carbohydrate and 56.58% protein recovery.
• Optimised US-DES extract showed higher fucose content and antioxidant capacity.
• FTIR and SEM confirmed chemical and structural changes after extraction.

## Graphical abstract

**Figure:**
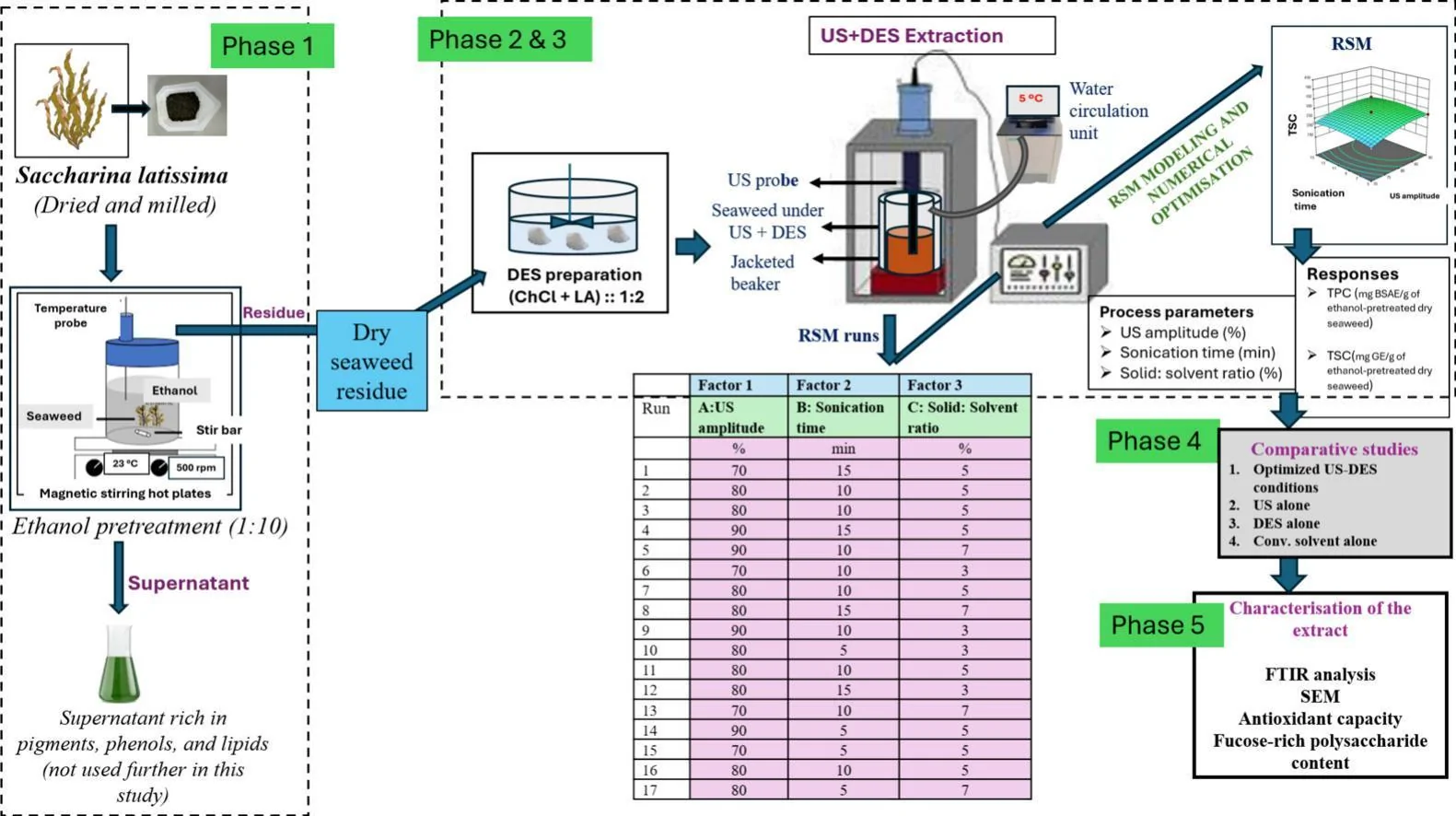

## 1 Introduction

Meeting the rising global need for proteins and bioactive carbohydrates sustainably signifies a critical challenge for the 21st century. Traditional protein crops, such as wheat, rice, maize, soy and other starch-based varieties, put forth immense pressure on freshwater, arable land and fertilisers, simultaneously causing significant levels of greenhouse gas emissions [1], [2], [3]. Of these, soy, a major plant-based protein source, has been reported to emit approximately 20.2 kg CO_2_-eq per kilogram of protein; however, this value is dependent on the specific geographical context, production system, system boundary, and methodological assumptions of the corresponding life-cycle assessment [4]. Considering these environmental pressures, marine seaweed, specifically *Saccharina latissima* (sugar kelp), a brown seaweed, emerges as a promising alternative. *S. latissima* can be cultivated in marine environments without direct requirements for agricultural land or freshwater irrigation, and seaweed cultivation can contribute to biological carbon uptake and nutrient removal from coastal waters. However, the extent to which these processes translate into durable carbon sequestration or net climate mitigation depends on cultivation conditions, biomass productivity, harvesting, transport, downstream processing and the subsequent fate of the biomass. Similarly, nutrient-removal benefits are site-specific and depend on local nutrient availability, hydrodynamics and cultivation practices. These considerations should therefore be evaluated within the specific production system rather than treated as universal environmental benefits [5], [6]. *S. latissima* is commonly used as food in Northern Europe and is available on the market in dried form from several seaweed enterprises as a source of bioactive compounds and minerals. Its biochemical profile has been extensively examined, with particular focus on carbohydrates, proteins, and other high-value compounds such as polyphenols and pigments, which are gaining extensive attention owing to their therapeutic properties [7], [8].

Environmental life-cycle assessment of farmed seaweed *S. latissima* protein production under Norwegian cultivation conditions, using a cradle-to-factory gate system boundary and 1 kg of crude protein as the functional unit, reported greenhouse gas emissions of 12.9 kg CO_2_-eq per kilogram [9]. The lowest value of 12.9 kg CO_2_-eq/kg crude protein was obtained under a scenario using surplus energy for drying, indicating that seaweed protein can have lower greenhouse gas emissions than the reported soy value under favourable production and processing conditions. However, these results are scenario-specific and depend on cultivation, processing and allocation assumptions. Considering the forecasted global protein demand to rise 10.5% from 2021 to 2028 [10], seaweed farming and its application signify a sustainable strategy for meeting nutritional macromolecule needs with decreased ecological impact. Beyond its environmental advantages, *S. latissima* is rich in both proteins and carbohydrates. The protein fraction varies from 3 to 12% of dry biomass, supplying necessary bioactive peptides and amino acids with functional benefits [11]. Carbohydrates, comprising alginate, laminarin, and fucoidan, possess immunomodulatory, antioxidative, and gelling behaviour, with wide relevance in food, pharmaceuticals, cosmetics, packaging, nutraceuticals, and so forth [12], [13], [14]. Co-recovery of proteins and carbohydrates is therefore essential for resource-efficient, economically viable seaweed biorefineries, enabling multiproduct valorisation of biomass and supporting circular bioeconomy strategies [5], [8].

To recover these valuable compounds, cutting −edge extraction approaches such as microwave, ultrasound, and high-pressure-assisted technologies have been utilised [15], [16]. These methods promote faster mass transfer and reduce processing time through cavitation, thermal, or pressure-facilitated cell lysis. Although effective, they are frequently combined with commonly used organic solvents or strong acids, which display substantial disadvantages: toxicity, volatility, flammability, environmental dumping concerns, and the challenge of degrading complex macromolecules [17], [18]. Water, being abundant and environmentally friendly, is now broadly applied as a solvent for protein and carbohydrate extraction. However, water still has limits, involving minimal selectivity and incomplete solubilization [19], [20]. Ionic liquids (ILs), such as 1-ethyl-3-methylimidazolium acetate ([EMIM][OAc]), 1-butyl-3-methylimidazolium chloride ([BMIM]Cl), 1-ethyl-3-methylimidazolium chloride ([EMIM]Cl) and so forth, have also been investigated as alternative extraction solvents because of their negligible vapour pressure, tunable physicochemical properties and strong solvation capacity. However, ILs constitute a highly diverse class of compounds, and their environmental, toxicological and regulatory profiles vary substantially according to their chemical structure. Moreover, most ILs are not GRAS (Generally Recognised as Safe), and given that the primary applications of seaweed proteins and carbohydrates lie in the food, feed and pharmaceutical sectors, their use is not commonly promoted in practice. Consequently, the suitability of a particular IL for extraction cannot be inferred solely from its regulatory classification or GRAS status. Practical considerations include ILs significant potential toxicity and ecotoxicity, residual solvent levels in the recovered product, synthesis, material costs, and recyclability [21]. These considerations are particularly relevant when extracts are intended for food, feed or nutraceutical applications [22], [23]. In response to these challenges, deep eutectic solvents (DESs) have been recognised as promising green solvent options. DES belong to a category of designer solvents synthesised through mixing a hydrogen bond acceptor (HBA) and a hydrogen bond donor (HBD) at a relative molar proportion to suppress the melting point, relative to each component alone [24], [25]. Also, DESs are considered “designer solvents” due to their tunability to tailor the extraction process. Thus, DESs aid targeted extraction under modest conditions, eliminating numerous challenges of organic and other extraction solvents. Nevertheless, their elevated viscosity and controlled mass transfer can decrease extraction efficiency [26], demanding careful process optimisation. Among the advanced extraction technologies available, ultrasound-assisted extraction can help overcome these limitations by generating acoustic cavitation, which produces microjets, shock waves, and localised shear forces that disrupt cell structures and enhance solvent penetration and mass transfer [1]. The mechanical effects of ultrasound can facilitate the release and diffusion of intracellular and cell-wall-associated compounds into the DES phase, while reducing the time required to achieve effective extraction. When combined with DES, ultrasound may therefore complement the solvent’s chemical solubilisation capacity by improving physical mass transfer and reducing the limitations associated with DES viscosity.

Although DES-assisted extraction shows considerable potential, there is a notable lack of research addressing its effectiveness in *S. latissima*. For instance, Moldes et al. [27] demonstrated selective extraction of protein from *S. latissima* using betaine and choline chloride-based DESs, yielding moderate recovery (∼10.6%) with minimal co-extraction of carbohydrate; Yet, process parameters for achieving good yields of both proteins and carbohydrates were not investigated, leaving co-extraction largely uninvestigated. Also, no advanced extraction technologies were used to improve the mass transfer. Jesus et al. [28] explored microwave-assisted DES extraction using DES formulation (sodium acetate:lactic acid and choline chloride (ChCl):lactic acid) for multiple valuable compounds from brown algae, accomplishing increased phenolic content; however, the extraction was performed at elevated temperatures (130–150 °C). The study also lacked systematic modelling and optimisation of the extraction process, a key step toward efficient seaweed biorefining. On the practical front, the linking of ultrasound-assisted (US) extraction with choline chloride-based DESs and optimisation by response surface methodology (RSM) has been demonstrated to be effective for flavonoid extraction from *Ilex asprella* [29]. Recently, Zin et al. [30] applied ultrasound-assisted deep eutectic solvent (US-DES) extraction using choline chloride/urea to microalgae proteins, exhibiting enhanced recovery of proteins and bioactivity. However, the variances in cell wall organisation and biochemical composition among microalgae and brown seaweed make translating these findings directly to seaweed biorefinery limited, particularly when targeting dual recovery of proteins and carbohydrates. Related studies have further demonstrated the potential of combining ultrasound with DES-based extraction for brown seaweed carbohydrates. Nie et al. [31] developed an ultrasound-assisted DES process for polysaccharide extraction from the brown seaweed *Sargassum horneri*, using a choline chloride:1,2-propanediol system and optimisation of solvent composition, water content, solid-to-liquid ratio and temperature. Although this study demonstrated the applicability of ultrasound-DES extraction to brown seaweed, it focused on polysaccharide recovery rather than simultaneous protein and carbohydrate recovery and used a different seaweed species and DES formulation. Similarly, Zeb et al. [32] investigated ultrasound-assisted extraction using water-rich natural DESs for polyphenol recovery from cultivated *S. latissima*. Their process was optimised using Box-Behnken response surface methodology and compared with conventional solvent extraction; however, the study targeted polyphenols rather than protein and carbohydrate fractions. More recently, Praveen et al. [33] combined ultrasound-assisted natural DES extraction with response surface methodology for the recovery of polysaccharides and phlorotannins from *Ecklonia radiata*, demonstrating the applicability of this integrated approach to brown seaweed bioactive recovery. Nevertheless, the target compounds, biomass and extraction objectives differed from those of the present study.

Together, these research studies uncover significant knowledge gaps: (i) the majority of studies emphasise either proteins or carbohydrates, but rarely simultaneously; (ii) advanced extraction techniques like ultrasound have been less exploited in DES-based seaweed extraction; (iii) systematic optimisation of the simultaneous recovery of proteins and carbohydrates from seaweed using DES-based extraction remains limited; and (iv) among various DES combinations, though (ChCl:lactic acid) offers low toxicity, high biodegradability, biocompatibility [34], and effective solubilisation of proteins and carbohydrates, yet it has not been applied for their simultaneous extraction from *S. latissima*. Alongside, comparative analyses against DES-only, ultrasound-only, and conventional aqueous extractions were conducted to evaluate the performance of the optimised US-DES treatment relative to the individual and conventional treatments. Accordingly, the present study addresses this gap by integrating ultrasound-assisted extraction with a ChCl-lactic acid DES and response surface methodology to optimise the simultaneous extraction of protein- and carbohydrate-rich fractions from *S. latissima*. Total protein content (TPC) and total soluble carbohydrate content (TSC) were used as the two primary responses for model development and numerical optimisation. Protein recovery yield (PRY) and carbohydrate recovery yield (CRY) were calculated from TPC and TSC, respectively, using the initial protein and carbohydrate contents of the ethanol-pretreated and dried biomass. Importantly, the study evaluates the combined process experimentally by direct comparison with conventional aqueous, ultrasound-only, and DES-only extraction under the conditions investigated.

## 2 Materials and methods

### 2.1 Biomass processing and chemical reagents

Dried and milled farmed *S. latissima* seaweed biomass was procured from Bantry Marine Research Station (Bantry, Ireland), and the biomass was stored in sealed containers at room temperature until further use. Before extraction, the biomass underwent an ethanol-based pretreatment to remove low-molecular-weight phenolics, lipids, and residual pigments, thereby minimising analytical interference and facilitating subsequent protein and carbohydrate recovery. As polyphenols, lipids, and pigments are highly soluble in ethanol, this pretreatment enabled their more effective removal and provided a cleaner biomass matrix for subsequent extraction and compositional analyses. The pretreatment conditions are as described. For the ethanol pretreatment, the procured powdered seaweed was blended with 80% (v/v) ethanol at a biomass-to-solvent ratio of 1:10 (w/v) and agitated for 24 h at 25 ± 1 °C using a magnetic stirrer set to 500 rpm. Subsequent to incubation, the blend was centrifuged (Beckman Coulter Avanti J-series centrifuge (Beckman Coulter, Brea, CA, USA)) for 30 min at 5000 × g, after which the recovered residue was oven-dried at 30 °C to constant weight (approximately 48 h), ensuring removal of residual ethanol. The dried material was subsequently re-milled and passed through a 100  µm mesh and stored in air-tight containers. The resulting ethanol-pretreated, dried, and re-milled biomass was used throughout the subsequent extraction and analytical procedures described in this study. Compositional analysis of ethanol-pretreated and dried seaweed was carried out, including the determination of moisture gravimetrically [2], protein was measured using the Kjeldahl method, with slight modifications [3] and a numerical value of 5 was used as a conversion factor for nitrogen [4], carbohydrate was measured using the Dubois method [5], fat was determined gravimetrically using the Soxhlet method [6], ash content was determined using a muffle furnace [2].

All chemicals and reagents used in this study were of analytical grade or the grade specified by the manufacturer. Ethanol (≥99.8%), choline chloride (≥99%), lactic acid (≥85%), sodium sulfite (≥98%), phenol (≥99%), sulfuric acid (95–98%), sodium hydroxide (≥98%), copper sulfate (≥98%), hydrochloric acid (37%), iron(III) chloride (≥97%), 2,4,6-tripyridyl-s-triazine (TPTZ; ≥99%), L-cysteine hydrochloride (≥98%), Triton X-100 (laboratory grade), methanol (≥99.8%), DPPH (≥95%), Trolox (≥97%), glucose (≥99%), fucose (≥99%), and ascorbic acid (≥99%) were purchased from Merck/Sigma-Aldrich (Dublin, Ireland). Bovine serum albumin (BSA; ≥98%) was obtained from Sigma-Aldrich (Dublin, Ireland). Milli-Q water was produced using a PURELAB Option water purification system (ELGA LabWater, Veolia Water Technologies, High Wycombe, UK) and was used for extraction and analytical procedures.

### 2.2 Synthesis of deep eutectic solvent (DES)

The DES used in this study was prepared by combining ChCl and lactic acid at a 1:2 M ratio, where ChCl served as the HBA and lactic acid acted as the HBD. The 1:2 M ratio was selected following preliminary evaluation of ChCl acid ratios of 1:1, 1:2, and 1:3. Among the formulations examined, the 1:2 mixture provided a homogeneous liquid and suitable protein and carbohydrate extraction performance under the preliminary test conditions. The ratio was therefore fixed for subsequent experiments to maintain a consistent solvent composition while investigating the effects of the extraction process parameters. Although increasing the proportion of lactic acid may modify the hydrogen-bonding environment and physicochemical properties of the solvent, a higher donor proportion was identified to lower the extraction performance, which might be owing to altered solvent interactions with the seaweed matrix. Henceforth, 1:2 M ratio was selected and kept constant. Milli-Q water produced using the PURELAB Option system (ELGA LabWater, Veolia Water Technologies, High Wycombe, UK) was incorporated into the DES. The components were mixed and gently heated to 60 °C for 1 h under continuous stirring until a homogeneous, clear liquid was obtained. Water was added at 40% (w/w) to reduce solvent viscosity and improve handling during extraction, following the approach reported previously [7], where this proportion was found to work best for bioactive compound extraction. The resulting aqueous DES mixture had a measured pH of approximately 2.5. This acidic environment may influence protein solubilisation and interactions between the solvent and seaweed matrix; However, the contribution of pH was not independently evaluated in this study. The mixture was then brought to ambient temperature (∼22 °C) and stored in a sealed container to prevent moisture absorption. All DES preparations were used on the same day to ensure consistency in extraction performance.

### 2.3 Ultrasound-assisted deep eutectic solvent (US-DES) extraction

The extraction was performed using a UIP500hdT ultrasonic processor (Hielscher Ultrasonics GmbH, Teltow, Germany) with a maximum output of 500  W. A titanium sonotrode (BS4d22) was immersed 30  mm into 200  mL of the pre-prepared seaweed-ChCl:lactic acid hydrated mixture (with varying solid-to-solvent ratio) and operated continuously. The ultrasonic processor was operated at a fixed frequency of 20 kHz, while the ultrasound intensity was varied through the amplitude setting. The frequency was kept constant based on the operating characteristics of the equipment, whereas amplitude was selected as an RSM variable because it directly controls the intensity of ultrasonic treatment. Preliminary single-factor trials were conducted to establish suitable ranges for ultrasound amplitude, sonication time, and solid-to-solvent ratio. Protein and total soluble carbohydrate contents were measured to assess extraction performance. Amplitude was initially examined over a range of 10–100%. Extraction improved with increasing amplitude. A marked increase in protein and carbohydrate content was observed from 70% amplitude. Above 90%, further increases in amplitude produced no significant improvement. Therefore, 70–90% was selected for the RSM design. Sonication time was varied in preliminary trials to identify the period required for effective extraction. Shorter treatment provided insufficient extraction, whereas longer treatment improved recovery up to a practical limit. Beyond this limit, no significant improvement was observed. Therefore, 5–15 min was selected. The solid-to-solvent ratio was also investigated in preliminary trials. Lower biomass loading provided greater solvent availability per unit mass of seaweed. Increasing biomass loading improved the amount of material processed per unit solvent but could restrict mixing and solvent access at higher ratios. Based on the observed extraction responses, a range of 3–7% was selected. The experimentally measured ultrasonic power output ranged from 213.7 ± 3.5 W at 70% amplitude for 5 min to 332.7 ± 6.5 W at 90% amplitude for 15 min. Temperature control was achieved using a jacketed beaker with a water circulation unit (Grant LT ecocool 100, Grant Instruments (Cambridge) Ltd., United Kingdom) maintained at 5 °C, and it was ensured that the maximum temperature of the solution did not exceed 30 °C during treatment. This was managed either by adding ice packs to the inlet tank of the water circulation unit or by introducing intervals between successive treatments, as required. The temperature was monitored using a digital thermocouple (G-TECH GT 305, Bangalore, India). This temperature limit was maintained to minimise thermal degradation or denaturation of proteins and preserve carbohydrate integrity, and to maintain consistent extraction conditions throughout the experiments, thereby supporting the co-extraction of both components. The DES composition, water content, pH and other extraction conditions were kept constant to isolate the effects of the three selected process variables. After sonication, the mixture was centrifuged at 4 °C at 10,000 × *g* for 30  min. The resulting extract-rich supernatant was retrieved and refrigerated at 4 °C for subsequent analyses.

### 2.4 Experimental design and mathematical modelling

The optimisation of US-DES extraction parameters for *S. latissima* protein- and carbohydrate-rich fractions was performed using response surface methodology (RSM) with a Box-Behnken Design (BBD). Three process parameters, including ultrasound amplitude (70–90%), sonication time (5–15 min), and solid-to-solvent ratio (3–7%), were investigated at three coded levels (−1, 0, +1) across 17 experimental runs, comprising five repeats at the central point, to assess their combined effects on the responses. The Box-Behnken experimental design comprising the three independent variables at their coded and actual levels is presented in Table 1. The optimisation was performed using two primary responses: TSC (Y1) and TPC (Y2). TSC and TPC represent the extracted soluble carbohydrate and protein amounts per unit mass of ethanol-pretreated dry biomass, respectively, and were therefore used as the primary responses for response-surface modelling and numerical optimisation. PRY and CRY were not included as independent optimisation responses because they are mathematically derived from TPC and TSC, respectively, using the fixed initial protein and carbohydrate contents of the biomass. PRY and CRY were instead calculated from the corresponding optimised and experimental TPC and TSC values to express recovery relative to the initial constituent content. Preliminary extraction experiments guided the selection of coded value limits. Table 1 summarises the parameter ranges for each factor in coded and actual units.

**Table 1:** Box-Behnken design showing coded and real variables of the extraction process.

Variable	Process parameter	Units	Minimum	Centre point	Maximum
			−1	0	+1
A	Ultrasound amplitude	%	70	80	90
B	Sonication time	Minute	5	10	15
C	Solid-to-solvent ratio	%	3	5	7

Each of the two primary responses, TSC and TPC, was modelled to a second-order polynomial regression fit:

(1) $$Y=\beta_{0}+\beta_{1}A+\beta_{2}B+\beta_{3}C+\beta_{11}A^{2}+\beta_{22}B^{2}+\beta_{33}C^{2}+\beta_{12}AB+\beta_{13}AC+\beta_{23}BC$$

where Y, represents the dependent response; $\beta_{0}$ is the intercept; $\beta_{1},\beta_{2},\beta_{3}$ are the linear coefficients; $\beta_{11},\beta_{22},\beta_{33}$ are the quadratic coefficients; and $\beta_{12},\beta_{13},\beta_{23}$ are the interaction coefficients. All experimental runs were performed in triplicate, and mean values were used for analysis. Statistical analyses, including analysis of variance (ANOVA), regression coefficients, model adequacy (R^2^, coefficient of variation, p-value), and lack-of-fit tests, were performed using Design-Expert software (Version 12, Stat-Ease Inc.). Three-dimensional response surface plots were generated to visualise the interactions between variables and their influence on each response. Numerical optimisation was performed using the desirability function approach, with total protein content (TPC) and total soluble carbohydrate content (TSC) as the two responses to be maximised simultaneously. The lower and upper limits for TPC were set at 9.972 and 51.730 mg BSAE/g ethanol-pretreated and dried biomass, respectively, while those for TSC were set at 170.844 and 435.504 mg GE/g ethanol-pretreated and dried biomass, respectively. Ultrasound amplitude, sonication time, and feed-to-solvent ratio were constrained to ranges of 70–90%, 5–15 min, and 3–7%, respectively. A weight of 1 and an importance value of 3 were assigned to each response. A weight of 1 specified a linear individual desirability function, while the equal importance values gave TPC and TSC equal relative priority in the overall optimisation. The individual desirability of each response ranged from 0 (unacceptable) to 1 (fully desirable), and the overall desirability was calculated as the geometric mean of the individual desirabilities. The optimal conditions were identified by maximising the overall desirability within the specified experimental ranges.

For a response to be maximised, the individual desirability function is:

(2) $$d_{i}=\left\{\begin{array}{cc} 0, & y_{i}<L_{i}\\ {\left(\frac{y_{i}-L_{i}}{U_{i}-L_{i}}\right)}^{w_{i}}, & L_{i}\leq y_{i}\leq U_{i}\\ 1, & y_{i}>U_{i} \end{array}\right)$$

where $y_{i}$ is the predicted response, $L_{i}$ and $U_{i}$ are its lower and upper limits, respectively, and $w_{i}$ is the desirability weight. With$w_{i}$ = 1, desirability increases linearly between the limits.

The overall desirability for the two equally important responses is:

(3) $$D=\sqrt{d_{TPC}\times d_{TSC}}$$

### 2.5 Validation and comparative experiments

To validate the RSM model, extractions were performed in triplicate under the predicted optimal US-DES conditions. *S. latissima* biomass was subjected to ultrasound-assisted extraction using the RSM optimised amplitude, sonication time, and solid-to-solvent ratio with ChCl-lactic acid DES. Extracts were analysed for the responses mentioned in section 2.4.

A comparative analysis was conducted to assess the performance of the different extraction treatments under their respective operating conditions. Four extraction approaches were tested: (i) Optimised US-DES, applying both ultrasound and DES under RSM optimal conditions; (ii) Ultrasound with conventional solvent (acidic water, pH 2.5 ± 0.1–0.2), maintained under the RSM optimal conditions but replacing DES with acidic water; (iii) DES without ultrasound, using ChCl: lactic acid (1:2 M ratio, containing 40% water) under RSM optimised conditions of solid-to-solvent ratio. An overhead stirrer (VOS 40 digital, VWR International Ltd., Dublin, Ireland) was employed to maintain continuous mixing of the mixture throughout the extraction for 90 min without sonication; and (iv) conventional solvent without ultrasound, employing acidic water (pH 2.5 ± 0.1–0.2) and continuous mixing for 90 min. Because the optimised US-DES treatment involved 12 min of sonication whereas the non-ultrasound treatments were conducted for 90 min, these comparisons were considered treatment-level performance comparisons rather than time-matched comparisons of individual process factors. Similarly, the ultrasound-only and US-DES treatments differ in solvent chemistry (acidic water versus ChCl acid DES). The 90-min duration for non-ultrasound treatments was selected to allow sufficient diffusion-driven solubilisation in the absence of cavitation. The pH 2.5 and temperature 30 ± 1 °C were kept constant across all four extraction methods to eliminate their influence on extraction performance. All comparative extractions were performed in triplicate. The four extraction methods were statistically compared using Tukey’s test performed in SPSS software.

### 2.6 Quantitative and qualitative analysis

#### 2.6.1 Determination of total protein content (TPC)

Total protein content (TPC) of the DES-treated seaweed extracts was determined using the Thermo Scientific Pierce BCA protein assay kit (obtained from Thermo Scientific, Rockford, IL, USA) following the manufacturer's instructions, as applied in previous studies [8]. Briefly, 20 μL of DES extract or BSA standard (0–2 mg/mL) was mixed with 980 μL of BCA working reagent (50:1 ratio of Reagent A: sodium carbonate, sodium bicarbonate, bicinchoninic acid, sodium tartrate in 0.1 M sodium hydroxide to Reagent B: 4% cupric sulfate) in a UV–Vis cuvette, giving a total reaction volume of 1 mL. The mixtures were thoroughly agitated for 30 s, incubated at 37 °C for 30 min, and then cooled to room temperature. Absorbance was measured at 562 nm using a UV–Vis spectrophotometer (Cary 60 UV–Vis, Agilent Technologies, Ketsch, Germany). Protein concentrations were expressed as mg bovine serum albumin equivalents (BSAE) per gram of ethanol-pretreated and dried seaweed. To validate the assay and assess potential interference from DES, five to six extracts were randomly selected from the 17 total runs for spike recovery experiments. Each extract was spiked with a known amount of Bovine Serum Albumin (BSA), and the rise in measured protein content was compared to the theoretical spike. The observed spike recovery ranged between 92–97%, which falls within the accepted limits for biological assays (typically 90–110%) [9]. Since the matrix interference was minimal, the standard protein estimation procedure was applied to all extracts.

#### 2.6.2 Determination of total soluble carbohydrate (TSC)

Total soluble carbohydrate (TSC) was quantified using the phenol–sulfuric acid method [5] with the interference-reduction pretreatment described earlier [10]. Briefly, aliquots of the liquid extracts were pretreated with sodium sulfite to quench oxidising impurities and minimise pigment interference as recommended by [10]. Subsequently, 143 µL of extract or glucose standard was combined with 714 µL of concentrated H_2_SO_4_, added all at once in a single step, then mixed thoroughly. This speeds up the reaction, stabilises the colour faster, ensures complete carbohydrate hydrolysis and gives more reproducible results, as reported by [10]. After mixing, 143 µL of 5% (w/v) phenol was added, and the reaction mixtures were incubated at 90 °C for 5 min and cooled to room temperature. Absorbance was measured at 490 nm using a UV–Vis spectrophotometer (Cary 60 UV–Vis, Agilent Technologies, Ketsch, Germany). d-Glucose calibration standards were prepared, and total soluble carbohydrate concentrations were quantified from the calibration curve and denoted as mg glucose equivalents (GE) per gram of ethanol-pretreated and dried seaweed. This assay detects all soluble carbohydrates, mainly including laminarin, fucoidan, partially soluble alginate, glucose, fucose, xylose, galactose and so forth present in *S. latissima* [11], [12]. Spike recovery tests, similar to those performed for TPC, were conducted for TSC and yielded values within the acceptable range, confirming minimal interference from DES.

#### 2.6.3 Carbohydrate recovery yield (CRY) and protein recovery yield (PRY)

Protein recovery yield (PRY) and carbohydrate recovery yield (CRY) were calculated from the corresponding TPC and TSC values, respectively, to express the recovered constituents as a percentage of their initial amounts in the ethanol-pretreated and dried seaweed. Accordingly, PRY and CRY were calculated using the following equations as described by [13]:

(4) $$PRY\;\left(\%\right)=\frac{C_{\mathrm{protein}}\times V_{\mathrm{extract}}}{10^{6}\times m_{\mathrm{biomass}}\times W_{\mathrm{protein}}}\times 100$$

(5) $$CRY\;\left(\%\right)=\frac{C_{\mathrm{carbohydrate}}\times V_{\mathrm{extract}}}{10^{6}\times m_{\mathrm{biomass}}\times W_{\mathrm{carbohydrate}}}\times 100$$

where $m_{\text{biomass}}$ is the dry weight of ethanol-pretreated and dried seaweed biomass (g), $V_{\text{extract}}$ is the total volume of the collected extract (mL), $C_{\text{carbohydrate}}$ and $C_{\text{protein}}$ are the calculated concentrations of carbohydrates and proteins in the liquid extract (µg/mL), and $W_{\text{protein}}$ and $W_{\text{carbohydrate}}$ are mass fractions of proteins and carbohydrates in the ethanol-pre-treated dried seaweed biomass, which were 0.0901 and 0.516, respectively. PRY and CRY represent normalised recovery indicators derived from the primary TPC and TSC responses and were not treated as statistically independent responses for modeling and optimisation.

#### 2.6.4 Antioxidant capacity assessment

##### 2.6.4.1 Ferric reducing antioxidant power (FRAP)

FRAP measurements were determined for US-DES extracts obtained under optimised conditions, and the extracts from the three comparative treatments (DES without ultrasound, ultrasound with conventional solvent, and conventional solvent without ultrasound) using the method explained by [14]. Fresh working FRAP reagent was prepared through combining acetate buffer (300 mM, pH 3.6) immediately before use, FeCl_3_ solution (20 mM), 2,4,6-Tripyridyl-s-triazine solution (10 mM in 40 mM HCl), and water in ratio 10:1:1:1.4 (v/v/v/v). For the assay, 50 µL of sample, blank, or Trolox standard was mixed with 950 µL of freshly prepared FRAP working solution. The reaction mixture was incubated at 37 °C in the dark for 30 min, and absorbance was recorded at 593 nm using a UV–Vis spectrophotometer (Cary 60 UV–Vis, Agilent Technologies, Ketsch, Germany) with a 1  mL quartz cuvette. The results were expressed as μM Trolox equivalents per mL of extract, based on standard curves prepared with Trolox.

##### 2.6.4.2 DPPH (2,2-Diphenyl-1-picrylhydrazyl) radical scavenging activity

DPPH assays were conducted on the US-DES extracts obtained under optimised conditions, and all comparative extractions applied the method described by [15]. A citrate–phosphate buffer (pH 5.0) containing 0.3% Triton X-100 was used to dissolve the samples, as well as the positive control (ascorbic acid). A total of 950  μL of extract solution or buffer was combined with 50 μL of 2 mM DPPH prepared in methanol, followed by a 30-minute incubation at room temperature in the absence of light. The value of absorbance was then noted at 515  nm using a 1  mL cuvette on a UV–Vis spectrophotometer. Radical scavenging activity (RSA) was estimated as the percentage DPPH inhibition relative to the blank, allowing comparison of antioxidant capacity among US-DES and the four control extractions.

(6) $$\%\;DPPH=\frac{Absorbance\;of\;blank-Absorbance\;of\;sample}{Absorbance\;of\;blank}\times \;\;100$$

#### 2.6.5 Fucose determination

Fucose content was determined colourimetrically and expressed as milligram (mg) fucose equivalents (FE) per gram (g) of ethanol-pretreated and dried seaweed. This measurement provides an estimate of fucose-containing material in the extract but does not constitute direct quantification or recovery determination of purified fucoidan. The estimation was performed according to the method described by [16] with the modifications previously stated by [1]. Samples, fucose standards (0.005–0.1 mg/mL), and a water blank were mixed with 4.5 mL of 80% sulfuric acid and heated at 100 °C for 10  min. After cooling, 0.1 ml of 3% L-cysteine hydrochloride was added, and the mixture was incubated for 60  min at room temperature. Absorbance was measured at 396 and 430  nm using a spectrophotometer (Epoch, BioTek, USA).

#### 2.6.6 Fourier transform infrared spectroscopy (FTIR)

FTIR analysis was carried out to characterise the functional groups present in the RSM-optimised US-DES liquid extract as well as in the three comparative extracts (DES without ultrasound, ultrasound with conventional solvent, and conventional solvent alone). Spectra were recorded using an FTIR spectrometer with an ID5 ATR accessory (iS50 Nicolet, Thermo Fisher Scientific, USA). For each measurement, a few drops of the liquid extract were placed directly onto the ATR crystal. Spectra were collected over a range of 4000–400  cm^−1^ at a resolution of 4  cm^−1^, with an average of 30 scans per sample. Ambient air spectra were used as background to correct for atmospheric contributions [17].

#### 2.6.7 Morphological analysis

The microstructural changes in the solid residue obtained after US-DES extraction under RSM-optimised conditions and residues from the comparative extractions (DES without ultrasound, ultrasound with conventional solvent, and conventional solvent alone) were analysed using scanning electron microscopy (SEM). The samples were mounted on aluminium SEM stubs with double sided carbon tape and sputter-coated with a 5 nm layer of gold/palladium (80:20) using a Quorum Q150 RES Sputter Coating System (Quorum Technologies, UK). Samples were examined using a Jeol JSM 5510 Scanning Electron Microscope (Jeol Ltd., Japan), using JSM-5000 software, Version 5.21. Observations were carried out at an acceleration voltage of 5  kV, spatial resolution of 10 µm and a magnification of 1000x.

### 2.7 Statistical analysis

All experimental data, including protein content, carbohydrate content, antioxidant capacity, and so forth for US-DES extracts and the comparative treatments. were conducted in triplicate unless noted otherwise, and data were processed using SPSS software (Version 22, SPSS Inc., Illinois, USA). Descriptive statistics, including means and standard deviations, were calculated for each parameter. To assess the accuracy and reliability of the developed response surface model, several statistical metrics were calculated, including average absolute deviation (AAD), mean percentage error (MPE), mean squared error (MSE), normalized mean squared error (NMSE), root mean squared error (RMSE), normalized root mean squared error (NRMSE), and the coefficient of determination (R2). These indicators collectively provided a comprehensive evaluation of the model's predictive performance (Eqs. (7), (8), (9), (10), (11), (12)).

(7) $$R^{2}=1-\frac{\sum {\left(y_{p}-y_{a}\right)}^{2}}{\sum {\left(y_{p}-y_{m}\right)}^{2}}$$

(8) $$\mathrm{MSE}=\frac{{\Sigma \left(y_{a}-y_{p}\right)}^{2}}{n}$$

(9) $$\mathrm{AAD}=\frac{\Sigma \left|y_{p}-y_{a}\right|}{n}$$

(10) $$\mathrm{NMSE}=\frac{\mathrm{MSE}}{{\frac{1}{n}\Sigma \left(y_{a}-y_{m}\right)}^{2}}$$

(11) $$\mathrm{RMSE}=\sqrt[{2}]{\mathrm{MSE}}$$

(12) $$\mathrm{MPE}=\frac{1}{n}\sum \left|\frac{y_{a}-y_{p}}{y_{a}}\right|*100$$

where *y_p_* is the predicted data value, *y_a_* are the actual data value, *y_m_* is the average experimental value and *n* is the number of experiments, respectively.

## 3 Result and Discussion

### 3.1 Proximate composition of *s. Latissima*

Table 2 presents the proximate composition (% dry weight) of ethanol-pretreated and dried seaweed, *S. latissima*. The biomass was characterised by dry matter (92.02 ± 0.08%) and relatively high ash content (19.29 ± 0.08%), reflecting its mineral-rich nature. Carbohydrates represented the major fraction (51.69 ± 1.01%), followed by protein (9.06 ± 0.22%) and low-fat content (1.98 ± 0.70%). Overall, the composition highlights *S. latissima* as a carbohydrate-rich and low-lipid biomass, supporting its investigation for the co-recovery of proteins and carbohydrates.

**Table 2:** Proximate composition (% dry weight basis) of ethanol-pretreated and dried seaweed (*S.latissima*).

**Nutrients**	**Composition**
Dry matter	92.02 ± 0.08
Ash	19.29 ± 0.08
Protein	9.06 ± 0.22
Carbohydrate	51.69 ± 1.01
Fat	1.98 ± 0.7

Values are presented as mean ± standard deviation (n = 3).

### 3.2 Model fitting

A Box-Behnken design (BBD) was employed to develop quadratic polynomial models describing the ultrasound-assisted deep eutectic solvent (US-DES) extraction of proteins and carbohydrates from *S. latissima*. Table 3 summarises the key statistical metrics, including AAD, MSE, MPE, RMSE, and NMSE for the studied experimental responses. The average experimental values and the RSM-predicted values of total protein content (TPC) and total soluble carbohydrates (TSC) from the 17 experimental runs under varying ultrasound amplitude, sonication time, and solid-to-solvent ratio are presented in Table 4. Second-order polynomial models were fitted to these responses, and their adequacy was evaluated through analysis of variance (ANOVA). The models for the two responses were highly significant (p < 0.0001), with coefficients of determination (R^2^) exceeding 0.98 (Table 5), indicating excellent predictive ability. Lack-of-fit tests were non-significant (p > 0.05) for the two responses (Table 6), confirming that the models accurately represent the experimental data. The models exhibited minimal error values as presented in Table 3 (MPE, AAD, MSE, NMSE, and RMSE), reflecting high predictive accuracy. Specifically, R^2^ values for TPC and TSC were 0.9810 and 0.9993, respectively, demonstrating excellent agreement between experimental and predicted values. Collectively, the low values of the calculated statistical performance metrics, together with the high R^2^ value, confirm the suitability of the developed models for the dataset.

**Table 3:** Performance metrics of the RSM model for the studied experimental responses.

**Responses**	**MSE**	**RMSE**	**NMSE**	**AAD**	**MPE**	**R^2^**
TSC	4.021	2.005	0.00066	1.170	0.006	0.9993
TPC	3.018	1.737	0.0190	1.458	0.116	0.9810

TSC: total soluble carbohydrates, TPC: total protein content, AAD: average absolute deviation, MPE: mean percentage error, MSE: mean squared error, NMSE: normalized mean squared error, RMSE: root mean squared error, NRMSE: normalized root mean squared error, and R^2^: coefficient of determination.

**Table 4:** The experimental and predicted values for total soluble carbohydrates and total protein content at various process parameter combinations.

Runs	Extraction parameters						
	Amplitude (%)	Sonication time (min)	Solvent: feed ratio (%)	TSC(mg GE/g of ethanol- pretreated and dried seaweed)		TPC (mg BSAE/g of ethanol- pretreated and dried seaweed)	
				Experimental value	RSM predicted value	Experimental value	RSM predicted value
1	70	15	5	226.5 ± 3.10	227.2	14.9 ± 0.40	13.5
2	80	10	5	283.4 ± 6.98	281.2	40.6 ± 1.38	38.3
3	80	10	5	283.8 ± 3.09	281.2	38.4 ± 0.77	38.3
4	90	15	5	275.7 ± 8.03	275.3	15.1 ± 0.30	17.4
5	90	10	7	205.3 ± 4.57	205.5	30.5 ± 0.88	27.8
6	70	10	3	364.1 ± 12.6	363.9	38.9 ± 0.97	41.5
7	80	10	5	274.1 ± 4.32	281.2	35.1 ± 0.81	38.3
8	80	15	7	195.7 ± 4.53	195.9	30.7 ± 0.61	31.0
9	90	10	3	435.5 ± 16.5	436.4	48.6 ± 0.97	47.8
10	80	5	3	399.3 ± 5.61	399.1	46.1 ± 0.93	46.1
11	80	10	5	282.1 ± 3.45	281.2	38.1 ± 0.98	38.3
12	80	15	3	413.1 ± 8.34	412.7	51.7 ± 1.03	50.5
13	70	10	7	170.8 ± 2.39	170.1	21.9 ± 0.44	23.0
14	90	5	5	272.8 ± 6.87	272.1	13.3 ± 0.54	14.8
15	70	5	5	211.8 ± 4.35	212.1	9.9 ± 0.34	7.6
16	80	10	5	282.8 ± 3.45	281.2	38.5 ± 0.77	38.3
17	80	5	7	190.7 ± 3.24	191.2	25.8 ± 0.52	27.0

TSC: total soluble carbohydrates, TPC: total protein content, GE: glucose equivalents, BSAE: bovine serum albumin equivalents.

**Table 5:** Regression coefficient, R^2^, Adjusted R^2^, Predicted R^2^ of second-order polynomial model for the responses’ total soluble carbohydrates and total protein content.

**Responses**	**Term**	**β coefficient**	**P value**
TSC model	$\beta_{0}$	281.24	< 0.0001
	$\beta_{1}$(A)	27.01	< 0.0001
	$\beta_{2}$(B)	4.56	0.0044
	$\beta_{3}$(C)	−106.19	< 0.0001
	$\beta_{12}$(AB)	−2.98	0.0985
	$\beta_{13}$(AC)	−9.24	0.0006
	$\beta_{23}$(BC)	−2.20	0.2023
	$\beta_{11}$(A^2^)	−20.16	< 0.0001
	$\beta_{22}$(B^2^)	−14.39	< 0.0001
	$\beta_{33}$(C^2^) CV: 1.11%	32.85	< 0.0001
	R^2^: 0.9993 Adj.R^2^: 0.9985 Pre.R^2^: 0.9984		

TPC model	$\beta_{0}$	38.25	< 0.0001
	$\beta_{1}$(A)	2.77	0.0233
	$\beta_{2}$(B)	2.12	0.0624
	$\beta_{3}$(C)	−9.63	< 0.0001
	$\beta_{12}$(AB)	−0.7891	0.5782
	$\beta_{13}$(AC)	−0.3479	0.8046
	$\beta_{23}$(BC)	−0.1089	0.9381
	$\beta_{11}$(A^2^)	−14.29	< 0.0001
	$\beta_{22}$(B^2^)	−10.64	< 0.0001
	$\beta_{33}$(C^2^) CV: 8.54%	11.04	< 0.0001
	R^2^: 0.9810 Adj.R^2^: 0.9565 Pre.R^2^: 0.7840		

TSC: total soluble carbohydrates, TPC: total protein content, CV: coefficient of variation, R^2^: coefficient of determination, Adj. R^2^: adjusted coefficient of determination, Pred. R^2^: predicted coefficient of determination

**Table 6:** Analysis of variance (ANOVA) of the developed RSM model for the studied experimental responses.

Source	Sum of squares	Degree of freedom	Mean square	F-value	p-value
**TSC**					
Model	1.034E + 05	9	11487.29	1175.71	< 0.0001
Residual	68.39	7	9.77		
Lack of Fit	3.39	3	1.13	0.0695	0.9733
Pure Error	65.01	4	16.25		
Cor Total	1.035E + 05	16			

**TPC**					
Model	2644.77	9	293.86	40.09	< 0.0001
Residual	51.30	7	7.33		
Lack of Fit	34.79	3	11.60	2.81	0.1719
Pure Error	16.51	4	4.13		
Cor Total	2696.07	16			

TSC: total soluble carbohydrates, TPC: total protein content.

### 3.3 Influence of extraction process parameters on total soluble carbohydrate (TSC)

The extraction of total soluble carbohydrates (TSC) from *S. latissima* using US-DES was strongly influenced by ultrasound amplitude, sonication time, and solid-to-solvent ratio, as demonstrated by a highly significant quadratic model (p < 0.001) with excellent predictive accuracy (R^2^ = 0.9993, adjusted R^2^ = 0.9985). The close agreement between experimental and predicted values (Table 4) further confirms the robustness and precision of the model in predicting carbohydrate solubilisation. The reduced regression equation, retaining only significant terms, was obtained as follows, where A is ultrasound amplitude, B is sonication time, and C is solid-to-solvent ratio:

(13) $$\mathrm{TSC}=281.24+27.01A+4.56B-106.19C-20.16A^{2}-14.39B^{2}+32.85C^{2}-9.24AC$$

The direction of the effects was determined by the sign of the regression coefficients (β), while statistical significance was evaluated using p-values. Among the independent parameters, ultrasound amplitude exerted the strongest positive linear effect (β_1_ = 27.01, p < 0.0001), while sonication time showed a statistically significant positive effect (β_2_ = 4.56, p = 0.0044) on TSC. In contrast, the solid-to-solvent ratio had a statistically significant negative linear effect (β_3_ = −106.19, p < 0.0001) on TSC, as shown in Table 5. Increasing ultrasound amplitude from 70% to approximately 85–90% resulted in a substantial rise in carbohydrate extraction, with the highest experimental TSC value of 435.5 ± 16.5 mg/g dry biomass obtained at 90% amplitude, 10 min sonication, and 3% (w/v) solid loading. This trend is clearly illustrated in the three-dimensional response surface plots (Fig. 1a–c). The increase in TSC with higher ultrasound amplitude can be attributed to intensified acoustic cavitation, which collapses violently, producing micro-jets and localised shear forces [18], [19]. These effects are particularly pronounced in brown seaweeds such as *S. latissima*, whose cell walls are rich in carbohydrates, forming a dense gel-like matrix [20], [21]. These findings are in agreement with previous studies, where ultrasound-assisted extraction significantly improved laminarin recovery from *Ascophyllum nodosum* and *Laminaria hyperborea* through cavitation-induced cell wall disruption and enhanced mass transfer [22]. This enhanced carbohydrate solubilisation can be rationalised by Kamlet-Taft parameters: lactic acid (α, π) forms hydrogen bonds with –OH, –COO⁻, and –OSO_3_⁻ groups, while choline chloride (β) stabilises charged moieties, weakening intra-and intermolecular interactions [23], [24]. Further, the wide range of soluble carbohydrates observed across the RSM runs may also be from partially depolymerised carbohydrates, oligosaccharides, rather than exclusively monosaccharides. Therefore, the value represents extractable carbohydrate equivalents rather than total chemical hydrolysis of all seaweed carbohydrates. Spike recovery tests with added glucose gave 90–97% recovery, confirming DES did not interfere with the assay. Thus, the elevated TSC results from genuine solubilisation of carbohydrates due to the combined chemical (–OH / C=O interactions) and physical (ultrasound) effects. Similar combined effects have been reported for ultrasound combined with choline-chloride-based DES during carbohydrate extraction from plant materials, where the chemical effect of DES disrupts hydrogen-bond networks and the physical effect of ultrasound promotes solute release [25], [26]. Despite the strong positive linear influence, a significant negative quadratic effect of amplitude (A^2^, $\beta_{11}$= −20.16, p < 0.0001) indicates a non-linear response, with the extraction response decreasing at higher amplitudes within the investigated range. Similar trends have been reported by [27]. This decline should be interpreted as an empirical response of the extraction system rather than direct evidence of carbohydrate degradation or chain scission. Possible contributing factors include changes in cavitation behaviour, viscosity, mass transfer, solvent accessibility or saturation, and experimental variability. Molecular-weight and detailed molecular characterisation would be required to determine whether structural changes or chain scission contribute to this behaviour and are therefore considered beyond the scope of the present study and for future investigation. Notably, in the present study, higher TSC was still achieved, likely due to strict temperature control (≤30 °C) and reduced DES viscosity, which together minimised degradation while maintaining efficient cavitation. Sonication time had a significant but comparatively weaker effect on TSC. Increasing it from 5 to 10–12 min improved extraction through prolonged cavitation and cell wall erosion, while further extension to 15  min caused a slight decline due to carbohydrate degradation or DES saturation (B^2^, p < 0.0001). Achieving high yields within short sonication times highlights the efficiency of US-DES compared to conventional thermal or microwave methods requiring longer durations and higher temperatures [28]. Solid-to-solvent ratio was the most influential factor affecting TSC. Reducing biomass from 7% to 3% markedly increased extraction across all amplitudes and sonication times (Fig. 1b and c). Lower solid loading enhanced diffusivity, improving ultrasonic propagation and mass transfer. In contrast, higher biomass increased slurry viscosity and limited solvent accessibility. This behaviour is well documented in DES-based extraction systems, where mass-transfer limitations at high solid loadings were a major bottleneck in the study performed by Ling and Hadinoto [29].

**Fig. 1:**
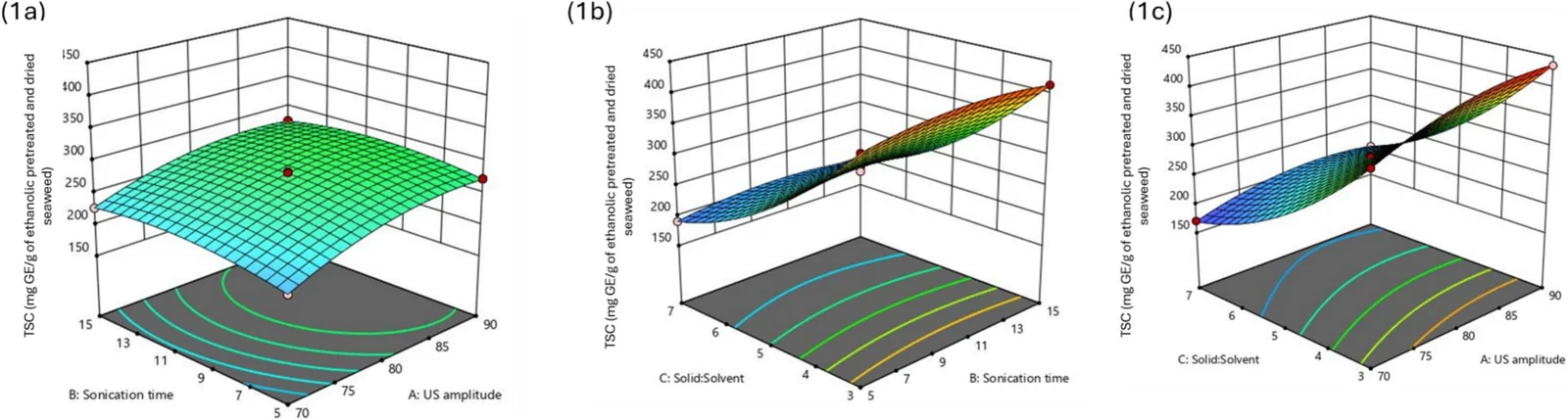
Three-dimensional response surface plot for total soluble carbohydrate from US-DES liquid seaweed extract as a function of significant interactions among factors: (1a) sonication time and ultrasound amplitude; (1b) solid-to-solvent ratio and sonication time;(1c) solid-to-solvent ratio and ultrasound amplitude.

With respect to interaction effects, the interaction between ultrasound amplitude and solid-to-solvent ratio (AC) was statistically significant (β_13_ = −9.24, p = 0.0006). The negative coefficient indicates that although increasing amplitude enhances TSC at low solid loadings, its beneficial effect diminishes as biomass concentration increases. This trend is clearly visible in Fig. 1c, where high amplitude combined with high solid loading resulted in a plateau or decline in TSC due to cavitation attenuation and limited solvent accessibility. Other interaction terms (AB and BC) were not significant (p > 0.05), indicating that sonication time did not strongly modulate the amplitude or solid loading effects on carbohydrate extraction. Overall, the TSC values obtained in this study were higher than those reported for the conventional DES extraction conditions considered here [30]. Compared with the microwave-assisted DES approach reported by Jesus et al. [28], which employed temperatures of 130–150 °C, the present US-DES process achieved high carbohydrate and protein recovery at 30 ± 1 °C. Thus, the present results demonstrate the effectiveness of US-DES as a low-temperature extraction approach under the conditions investigated. However, a direct comparison of energy consumption, sonication time, biomass loading, solvent composition, and target compounds was not performed and is beyond the scope of the present study.

### 3.4 Derived carbohydrate recovery yield (CRY)

CRY represents the proportion of the initial carbohydrate content recovered during extraction and was calculated directly from TSC. Accordingly, CRY follows the same trends as TSC, and their response-surface trends follow those of the corresponding primary response. Accordingly, the trends observed for CRY are consistent with those presented for TSC in Fig. 1(a–c). The results showed that ultrasound amplitude and sonication time positively influenced carbohydrate recovery, whereas increasing the solid-to-solvent ratio reduced extraction efficiency. For example, at 3% solid loading and 10 min sonication, increasing the ultrasound amplitude from 70% to 90% increased the derived CRY. Similarly, at 80% amplitude and 3% solid loading, increasing sonication time from 5 to 15 min also increased CRY. These trends are consistent with enhanced cavitation, solvent penetration, and mass transfer at higher ultrasound amplitudes and longer sonication times, while increased biomass loading may limit solvent availability and extraction efficiency [31], [32]. Also, derived CRY decreases sharply with increasing solid-to-solvent ratio across the investigated sonication times, identifying solvent availability as a key limiting factor, an effect well documented in DES- and ionic-liquid-based carbohydrate extraction systems [29], [33]. Beyond physical effects, the high CRY achieved under US-DES conditions is strongly underpinned by molecular-level interactions between the DES and seaweed carbohydrates. Alginate contains abundant carboxylate (–COO^–^) and (–OH) groups, laminarin is rich in β-glucan –OH functionalities, and fucoidan possesses (–OSO_3_^-^) groups. In the ChCl:lactic acid DES, lactic acid acts as an HBD, forming strong hydrogen bonds with (–OH) and (–COO^–^) groups, while ChCl functions as an HBA, interacting electrostatically with carboxylate and sulfate moieties. These interactions weaken inter- and intramolecular cross-linking within the carbohydrate network, facilitating solubilisation [34]. Ultrasound further enhances this effect by exposing additional functional groups through cavitation-induced micro-fracturing, resulting in accelerated mass transfer and high recovery yields. Similar synergistic mechanisms have been reported from plant materials [26], [35]. Overall, CRY provides a percentage-based interpretation of the carbohydrate recovery reflected by TSC, supporting the assessment of the optimised extraction process without requiring a separate RSM model.

### 3.5 Influence of extraction process parameters on total protein content (TPC)

The TPC under US-DES conditions exhibited a distinct response behaviour relative to carbohydrate extraction, reflecting the lower abundance, structural sensitivity, and strong associations of proteins with carbohydrates networks within the seaweed matrix. The quadratic response surface model for TPC was statistically significant (p < 0.0001) and demonstrated reliable predictive performance (R^2^ = 0.9810, adjusted R^2^ = 0.9565), with model error (RMSE = 1.737 mg/g) and coefficient of variation (CV = 8.54%) within acceptable limits for biological extraction systems. The reduced regression equation for TPC, containing only statistically significant terms, was:

(14) $$\text{TPC}\;\left(\text{mg BSAE}/\text{g}\right)\;=\;\text{38}.\text{25}\;+\;\text{2}.\text{77A}\;-\;\text{9}.\text{63C}\;-\;\text{14}.{\text{29A}}^{\text{2}}-\;\text{1}0.{\text{64B}}^{\text{2}}+\;\text{11}.0{\text{4C}}^{\text{2}}$$

Among the independent parameters, ultrasound amplitude showed a significant positive linear effect on TPC (p = 0.0233). Increasing amplitude to ∼ 80–85% enhanced protein extraction, with the highest experimental TPC of 51.7 ± 1.03 mg BSAE/g obtained at 80% amplitude, 15 min sonication, and 3% solid loading (Table 4). This improvement can be attributed to ultrasound-induced disruption of protein-carbohydrate complexes and enhanced penetration of the DES into intracellular regions, consistent with reports where moderate cavitation improved protein accessibility in algal and plant matrices as reported previously [36], [37]. Across the US-DES experiments, TPC exhibited a wide variation ranging from ∼ 9 to 51.7 mg BSAE/g of ethanol-pretreated and dried seaweed, reflecting the strong sensitivity of protein solubilisation to process conditions. The ChCl:lactic acid DES used in this study (40% water) had an acidic pH of ∼ 2.5, under which intact seaweed proteins would typically show low solubility due to aggregation near or below their isoelectric region. Accordingly, TPC values below ∼ 39 mg BSAE/g of ethanol-pretreated and dried seaweed observed mainly at high solid loadings (≥5%) or excessive ultrasound energy can be attributed to limited protein desorption arising from high slurry viscosity, carbohydrate shielding, and insufficient cavitation penetration. In contrast, under optimised conditions (3)% solids, 80–85% amplitude, moderate sonication), TPC increased markedly (39–51.7 mg BSAE/g ethanol-pretreated and dried seaweed) despite the same acidic pH, indicating that protein extraction was governed primarily by matrix disruption rather than pH alone. Ultrasound-induced cavitation, together with extensive soluble carbohydrate removal, exposed previously inaccessible protein domains and promoted the release of partially unfolded proteins and peptide-rich fractions. At the molecular level, lactic acid forms hydrogen bonds with amide (–CONH⁻) and amino (–NH_2_) groups, while choline chloride stabilises exposed polar and charged residues via electrostatic and dipolar interactions, enabling protein dispersion and solubilisation through electrostatic and dipolar interactions even under acidic conditions [37]. In agreement with this, a strong negative quadratic effect of amplitude (p < 0.0001) indicated that further increases in ultrasound intensity reduced TPC, as shown in Fig. 2a. Excessive cavitation likely induces protein unfolding, aggregation, or partial denaturation, reducing solubility in the DES phase, a phenomenon widely observed for ultrasound-assisted protein extraction at high energy inputs [36]. Sonication time exhibited a marginal positive linear effect (p = 0.06) but a significant negative quadratic effect (p < 0.0001), with moderate sonication promoting protein release and prolonged exposure reducing extractable protein, as illustrated in Fig. 2b. Beyond physical effects, DES chemistry played a critical role in seaweed protein solubilisation. In the ChCl: lactic acid DES, lactic acid also interacts with protein amide carbonyl (–C=O) groups; these interactions weaken protein-carbohydrate and protein-cell wall associations, improving solubility under moderate ultrasound conditions. Ultrasound further enhances this mechanism by exposing buried functional groups through micro-fracturing of the matrix. Solid-to-solvent ratio exerted a strong negative linear influence on TPC (p < 0.0001), with lower biomass loadings enhancing protein extraction by reducing viscosity and competition for solvent hydrogen-bonding sites. This is evident in Fig. 2c, where TPC is highest at 3% solids and declines at greater loadings, a behaviour attributed to increased solution viscosity and carbohydrate shielding of proteins at higher biomass concentrations. Unlike carbohydrate extraction, all interaction terms (AB, AC, BC) for TPC were non-significant (p > 0.05), indicating that protein extraction was mainly governed by independent effects of ultrasound amplitude, sonication time, and solid loading. Response surface trends show that protein yield increased with optimised amplitude or time individually, while combined high amplitude and prolonged sonication did not enhance TPC, likely due to protein destabilisation. This additive behaviour is consistent with prior ultrasound assisted extraction studies, where independent parameter optimisation was more critical than interaction effects [36], [37]. The strong linkage between carbohydrate removal and protein accessibility highlights the importance of balancing carbohydrate solubilisation and protein stability to maximise protein recovery from *S. latissima*.

**Fig. 2:**
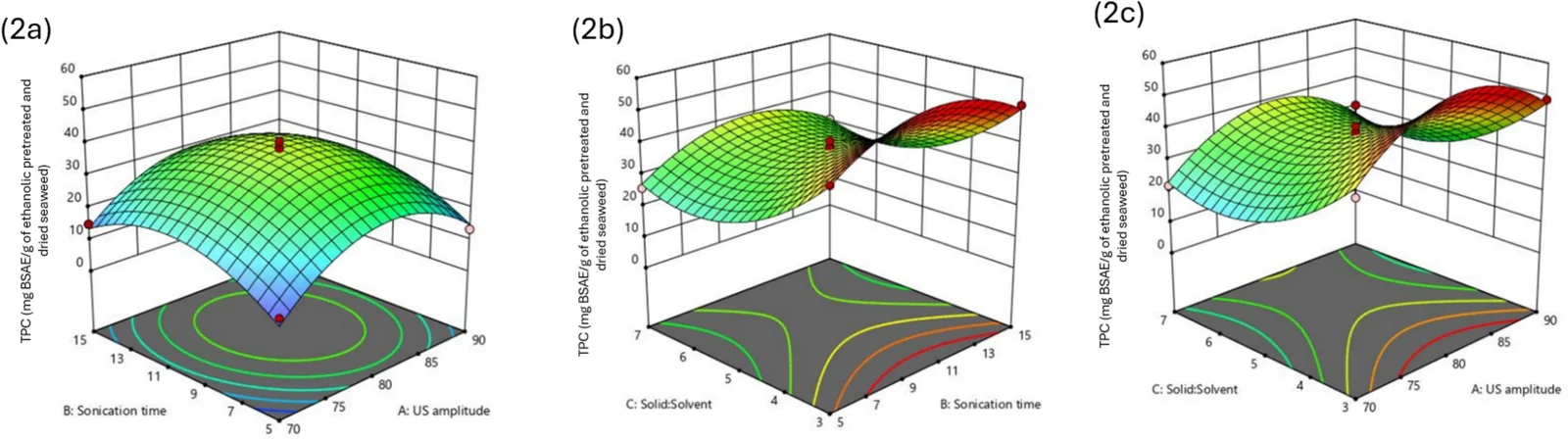
Three-dimensional response surface plot for total protein content from US-DES liquid seaweed extract as a function of significant interactions among factors: (2a) sonication time and ultrasound amplitude; (2b) solid-to-solvent ratio and sonication time; (2c) solid-to-solvent ratio and ultrasound amplitude.

### 3.6 Derived protein recovery yield (PRY)

PRY reflects the efficiency of protein extraction relative to the initial protein content of the biomass and was calculated directly from TPC. Accordingly, PRY follows the same trends as TPC, and their response-surface trends follow those of the corresponding primary response. Accordingly, the trends observed for PRY are consistent with those presented for TPC in Fig. 2(a–c). Increasing ultrasound amplitude generally enhanced protein recovery, consistent with improved cavitation, cell disruption, and mass transfer, although excessive ultrasound intensity may adversely affect protein solubility through denaturation or aggregation [36], [37]. Sonication time also influenced recovery, while increasing the solid-to-solvent ratio reduced extraction efficiency, likely because of restricted solvent accessibility and mass transfer at higher biomass loadings. The ChCl acid DES may facilitate protein solubilisation through hydrogen-bonding and electrostatic interactions, while ultrasound can promote the release of proteins from the seaweed matrix. These mechanisms are plausible explanations for the observed extraction trends but were not directly established in this study. The non-significant interaction effects previously observed in the four-response analysis are not discussed further because PRY is derived from TPC rather than independently modelled. The maximum PRY of 57.61% achieved in this study indicates substantial recovery of the initial protein content under the optimised US-DES conditions. This value exceeds protein recovery yields reported for S. latissima using conventional approaches, including approximately 16.0% following alkaline extraction and acid precipitation [38]. Similarly, pH-shift-based methods produced up to 25.1% protein yield from *S. latissima* biomass [39] and aqueous or enzymatic extraction procedures generally report yields well below 30% [40]. These comparisons suggest that the US-DES approach is promising for protein recovery; however, differences in extraction protocols and yield definitions should be considered when comparing results across studies. Overall, PRY provides a percentage-based interpretation of the protein recovery reflected by TPC, supporting the assessment of the optimised extraction process without requiring a separate RSM model.

### 3.7 Optimisation of US-DES extraction parameters for *s. Latissima*

The numerical optimisation identified optimal extraction conditions of 88% ultrasound amplitude, 12 min sonication time, and a solid-to-solvent ratio of 3%, yielding predicted TSC and TPC values of 435.96 mg GE/g and 51.91 mg BSAE/g ethanol-pretreated and dried biomass, respectively. Both predicted responses slightly exceeded their respective upper desirability limits of 435.50 mg GE/g for TSC and 51.73 mg BSAE/g for TPC. Accordingly, both individual desirability values reached the maximum of 1.00, resulting in an overall desirability of 1.00. Experimental validation under the optimised conditions yielded 431.09 ± 17.48 mg GE/g ethanol-pretreated and dried biomass for TSC and 50.98 ± 1.29 mg BSAE/g ethanol-pretreated and dried biomass for TPC, supporting the suitability of the optimised conditions for the simultaneous extraction of soluble carbohydrates and protein from *S. latissima.*

### 3.8 Verification of the model

The predicted, derived and experimentally validated responses are presented in Table 7. Validation experiments were conducted in triplicate using the above optimised conditions, and the results are presented in Table 7. The experimental data closely matched the values predicted by the response surface model and derived from the primary predicted response values, with deviations within the measured standard error. This close correspondence demonstrates the accuracy and robustness of the RSM model for multi-response optimisation.

**Table 7:** Predicted and validated values of response using RSM-optimised ultrasound-deep eutectic solvent (US-DES) conditions.

Optimized conditions	RSM predicted primary responses	Derived recovery yield	Validated responses
US amplitude − 88% US time − 12 min Solid: solvent – 3%	TSC: 435.96 mg GE/g ethanol-pretreated dried seaweed TPC: 51.91 mg BSAE/g ethanol- pretreated dried seaweed	CRY: 84.48% PRY: 57.61%	TSC: 431.09 ± 17.48 mg GE/g ethanol-pretreated dried seaweed CRY: 83.54 ± 3.18% TPC: 50.98 ± 1.29 mg BSAE/g ethanol-pretreated dried seaweed PRY: 56.58 ± 1.39%

TSC: total soluble carbohydrates, CRY: carbohydrate recovery yield, TPC: total protein content, PRY: protein recovery yield, GE: Glucose equivalents, BSAE: Bovine serum albumin equivalents; US: ultrasound.

### 3.9 Comparative evaluation of extraction strategies

The comparative evaluation of extraction strategies demonstrates a clear, stepwise enhancement in targeted responses from *S. latissima* as physical and chemical intensification mechanisms are progressively combined (Table 8). Conventional extraction using acidic water (pH ∼ 2.5 ± 0.1–0.2) at 30 °C resulted in very low yields across all responses, reflecting the limited solubilisation capacity of acidic water alone and the highly ordered carbohydrate-protein network of brown seaweed cell walls. At acidic pH, proteins approach or fall below their isoelectric region, promoting aggregation, while the dense carbohydrate matrix restricts solvent penetration and mass transfer. Comparable studies on aqueous or mildly acidic extraction of *S. latissima* report similarly low carbohydrate (<100 mg/g) and protein (<10%) recoveries [41], [42]. Applying ultrasound in acidic water (US alone) significantly improved all responses due to cavitation-induced disruption of the seaweed matrix and enhanced mass transfer. However, protein recovery remained low compared with ultrasound-assisted extractions conducted in neutral or alkaline media, where protein yields of ∼ 20–35% have been reported for seaweed [43], [44]. This confirms that the acidic solvent, rather than ultrasound efficiency, constrained protein solubilisation, whereas carbohydrate extraction benefitted more strongly because fucoidan and laminarin exhibit enhanced solubility under acidic conditions. Carbohydrate recoveries obtained under low-temperature ultrasound conditions in the present study are comparable to those reported in ultrasound-assisted extractions performed at similar temperature ranges (∼23–30 °C) [1]. However, higher carbohydrate yields have been reported when thermally intensified techniques such as microwave extraction or high-temperature ultrasound (>90 °C) were applied, reflecting the strong temperature dependence of laminarin and fucoidan solubility [1]. DES-only extraction using ChCl:lactic acid (40% water) further increased extraction efficiency, highlighting the strong chemical contribution of the solvent system. Comparable DES-based studies on *Dioscorea opposita* report carbohydrate recoveries of ∼ 30–50%, consistent with these results [45]. The effectiveness of this DES can be rationalised using Kamlet-Taft parameters: choline chloride-based DES exhibit high hydrogen bond basicity (β), while lactic acid provides strong hydrogen bond donating ability (α) and moderate dipolarity/polarisability (π*). Lactic acid forms hydrogen bonds with carbohydrates –OH, –COO⁻, and –OSO_3_⁻ groups, while choline chloride stabilises peptide backbone carbonyls (–C=O), amide (–CONH–) and charged side chains, weakening carbohydrate-protein associations and enabling solubilisation. The optimised US-DES treatment produced the highest values across all measured responses, indicating enhanced extraction performance under the combined ultrasound-DES conditions. These carbohydrate recoveries exceed those typically reported for ultrasound and dynamic maceration extraction of Arctic brown algae, where CRY often remains below ∼ 50–60% even at high energy input [46]. Mechanistically, ultrasound generates micro-fractures and exposes buried functional groups, while the DES disrupts hydrogen-bond networks and stabilises solubilised carbohydrates and peptide-rich protein fractions. Extensive carbohydrate removal further reduces carbohydrate shielding, directly enhancing protein accessibility and linking high CRY to improved PRY. Overall, the integrated US-DES strategy provided substantially higher carbohydrate and protein recovery than the individual treatments under the respective experimental conditions.

**Table 8:** Comparative evaluation of targeted responses for RSM-optimised US-DES extraction condition against conventional, ultrasound-only, and DES-only methods.

**Responses**	**Extraction method**			
	Conventional	US alone	DES alone	Optimized US-DES
TSC (mg GE/g ethanol-pretreated dried seaweed)	54.17 ± 2.79^a^	132.48 ± 6.67^b^	164.42 ± 13.09^c^	431.00 ± 17.48^d^
CRY (%)	10.49 ± 0.54^a^	25.67 ± 1.18^b^	31.86 ± 2.53^c^	83.52 ± 3.38^d^
TPC (mg BSAE/g ethanol-pretreated dried seaweed)	5.15 ± 0.14^a^	8.89 ± 0.37^b^	25.14 ± 0.87^c^	50.98 ± 1.29^d^
PRY (%)	5.72 ± 0.17^a^	9.87 ± 0.41^b^	27.90 ± 0.96^c^	56.60 ± 1.43^d^

CRY and PRY were calculated from TSC and TPC, respectively, using the initial carbohydrate and protein contents of the ethanol-pretreated biomass.

Values are expressed as mean ± SD (n = 3). Different superscript letters within the same row indicate significant differences among extraction methods according to Tukey’s test (p < 0.05).

TSC: total soluble carbohydrates, CRY: carbohydrate recovery yield, TPC: total protein content, PRY: protein recovery yield, GE: Glucose equivalents, BSAE: Bovine serum albumin equivalents, US: ultrasound, DES: deep eutectic solvent; US-DES: ultrasound-deep eutectic solvent.

The growing application of ultrasound-assisted deep eutectic solvent (US-DES) systems in the recovery of bioactive constituents from seaweeds further supports the relevance of this integrated extraction approach. Recent studies further demonstrate the growing application of ultrasound-assisted deep eutectic solvent (UL-DES) systems for the recovery of bioactive compounds from seaweed and other biomasses. Olfat et al. [47] applied ultrasound-assisted natural deep eutectic solvent extraction to *Hypnea flagelliformis* using a choline chloride acid-based NADES and reported enhanced recovery of bioactive and phenolic compounds together with antioxidant capacity compared with conventional solvents. Hu et al. [48] optimised ultrasound-assisted DES extraction of carbohydrates from *Porphyra haitanensis* using choline chloride (1:1), 60% water, 200 W, 120 min and 80 °C, achieving an extraction yield of 4.42%. Zeb et al. [49] investigated ultrasound-assisted water-rich NADES extraction of polyphenols from Saccharina latissima, with optimized conditions of 50 °C, 1 h and a 1:20 (w/w) biomass-to-solvent ratio, obtaining 15.97 mg GAE/g of recovered polyphenolic extract. Compared with these recent studies, the present work focuses on the simultaneous recovery of proteins and carbohydrates from S. latissima using ChCl acid DES under comparatively mild conditions of 30 ± 1 °C and a short sonication time of 12 min. However, differences in seaweed species, biomass composition, DES formulation, water content, extraction temperature and time, target compounds, and analytical methods prevent direct quantitative ranking of the reported extraction performances. These studies therefore provide contextual evidence of the versatility of US-DES approaches, while the present study extends their application to the simultaneous recovery of protein and carbohydrate fractions from S. latissima.

### 3.10 Effect of extraction technologies on antioxidant capacity and fucose content

The chemical antioxidant capacity of the crude extracts was evaluated using DPPH radical-scavenging and FRAP assays. The antioxidant potential of liquid extracts from *S. latissima* was strongly influenced by extraction methodology (Table 9). The optimised US-DES extraction achieved the highest DPPH radical scavenging activity (73.5 ± 1.3%) and FRAP (134.5 ± 1.67 µM TE/ml extract), surpassing DES alone (65.2 ± 0.95%; 87.8 ± 2.53 µM TE/ml), US alone (59.1 ± 1.04%; 90.3 ± 1.18 µM TE/ml), and conventional acidic water (23.5 ± 0.67%; 19.5 ± 0.74 µM TE/ml). These results indicate greater chemical antioxidant capacity of the crude US-DES extract compared with the other extraction treatments. However, because the assays were performed on crude extracts, the measured antioxidant response cannot be assigned specifically to carbohydrates or proteins and may reflect the combined contribution of co-extracted constituents. Nevertheless, the potential contribution of pigments and polyphenolic compounds is expected to be reduced, as these constituents were removed from the biomass to a considerable extent during the ethanol pretreatment. Therefore, the observed antioxidant capacity may be primarily associated with the carbohydrate- and protein-rich constituents, while a contribution from other co-extracted compounds cannot be completely excluded. Interestingly, a slightly higher FRAP value observed for US-only extracts relative to DES-only extracts is likely due to ultrasound-enhanced release of low-molecular-weight reducing sugars and peptide fragments, which contribute more effectively to ferric ion reduction than the predominantly sulphated carbohydrates enriched under DES conditions. The fucose content was highest in the optimised US-DES extract (20.56 ± 0.46 mg FE/g of ethanol-pretreated and dried seaweed), followed by DES alone (13.14 ± 0.22 mg FE/g g of ethanol-pretreated and dried seaweed), US alone (4.65 ± 0.09 mg FE/g g of ethanol-pretreated and dried seaweed), and conventional extraction (0.95 ± 0.02 mg FE/g g of ethanol-pretreated and dried seaweed). These results indicate greater recovery of fucose-containing material under the combined US-DES conditions. However, because the assay measures fucose equivalents rather than purified fucoidan, the result should be interpreted as an indication of enrichment in fucose-containing carbohydrate fractions rather than direct fucoidan recovery. Molecular and compositional characterisation of the recovered carbohydrate fractions would be required to confirm their identity and structural composition. The higher recovery observed here can be attributed to the combined action of ultrasound-induced matrix disruption and the strong hydrogen-bond donor–acceptor capacity of the ChCl:lactic acid DES, which promotes selective solubilisation of sulphated fucoidan even after ethanol pre-treatment. Comparatively, ultrasound-microwave-assisted brown seaweed extracts report DPPH ∼ 80–90% RSA [1], [44], slightly higher than the present US-DES extracts, likely due to phenolic retention in those studies, which were largely removed during our ethanol pre-treatment. Overall, the data demonstrate that the integrated US-DES treatment produced the highest fucose-rich carbohydrate recovery and antioxidant capacity under the selected treatment conditions, while conventional acidic extraction was highly limited in efficacy. Because the treatment durations were not time-matched, these results are interpreted as comparative treatment performance rather than quantitative evidence of synergy.

**Table 9:** Comparative evaluation of DPPH, FRAP, and fucose content for RSM-optimised US-DES extraction conditions against conventional, ultrasound-only, and DES-only methods.

**Responses**	**Extraction method**			
	Conventional	US alone	DES alone	Optimized US + DES
DPPH (%RSA)	23.46 ± 0.67^a^	59.14 ± 1.04^b^	65.23 ± 0.95^c^	73.46 ± 1.3^d^
FRAP (µM TE/ml of extract)	19.48 ± 0.74^a^	90.32 ± 1.18^b^	87.81 ± 2.53^b^	134.48 ± 1.67^c^
Fucose content(mg FE/g of ethanol- pretreated and dried seaweed)	0.95 ± 0.02^a^	4.65 ± 0.09^b^	13.14 ± 0.22^c^	20.56 ± 0.46^d^

%RSA: percentage radical scavenging activity, TE: trolox equivalents, FE: fucose equivalents.

Values are expressed as mean ± SD (n = 3). Different superscript letters within the same row indicate significant differences among extraction methods according to Tukey’s test (p < 0.05).

DPPH: (2,2-Diphenyl-1-picrylhydrazyl), FRAP: Ferric reducing antioxidant power, US-DES: ultrasound-deep eutectic solvent.

### 3.11 FTIR characterisation of extracts

FTIR spectra were used to confirm the functional groups of proteins and carbohydrates extracted from *S. latissima* and to distinguish them from solvent contributions (Fig. 3). The DES baseline (3a) exhibited characteristic bands at ∼ 3350 cm^−1^ (O–H stretching), ∼2987 cm^−1^ (C–H stretching of choline), ∼1723 cm^−1^ (C=O stretching of lactic acid), and ∼ 1046 cm^−1^ (C–O stretching), consistent with choline chloride-lactic acid systems [50]. Upon extraction, both DES-alone (3c) and US-DES extracts (3b) showed additional bands at ∼ 1635 cm^−1^ corresponding to Amide I of proteins, ∼1234 cm^−1^ assigned to sulfate (S=O) stretching of fucoidan, broad absorption band observed at ∼ 3300 cm^−1^ for spectrum of US-DES, DES, and US compared to DES baseline is attributed to O–H stretching vibrations of β-glucan hydroxyl groups from laminarin, with contribution from hydrogen-bonded and adsorbed water and weak bands at ∼ 840–850 cm^−1^ indicative of sulphated carbohydrates. These bands were markedly more intense in the US-DES extract, confirming enhanced solubilisation of proteins and carbohydrates via combined cavitation and DES-biopolymer interactions, without formation of new chemical functionalities. In the water system (pH 2.5), the baseline spectrum (3d) showed only a broad O–H band. Extracted samples (3e-f) displayed similar protein and carbohydrate peaks, though with lower intensity than DES-based systems, indicating reduced extraction efficiency. Overall, peak intensity followed the following trend: US-DES > DES > US-water > Conventional, validating quantitative extraction results.

**Fig. 3:**
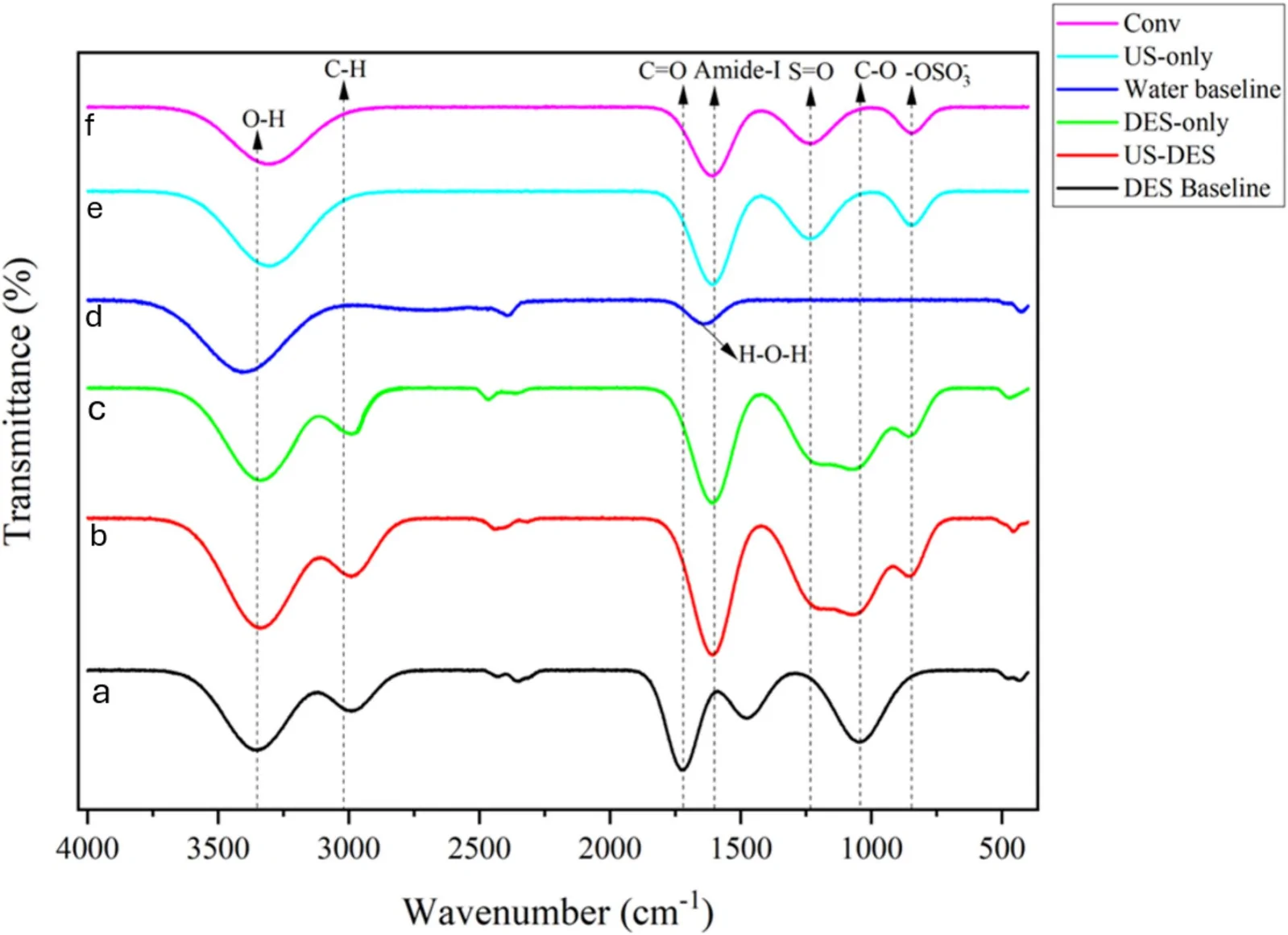
FTIR spectra showing the DES baseline (3a), acidified water baseline (3d), and extracted samples: optimal US-DES (3b), DES alone (3c), US alone (3e), and conventional (3f) extraction.

### 3.12 Morphological changes in seaweed residues after extraction

Scanning electron microscopy (SEM) images recorded at 1000 × magnification (Fig. 4a–e) illustrate the progressive disruption of *S. latissima* cell wall architecture under different extraction treatments, in line with the observed extraction efficiencies. The biomass following ethanol pretreatment and subsequent drying, with no further extraction treatment (Fig. 4a), shows a compact, continuous surface with intact lamellae, reflecting a dense, carbohydrate-rich matrix dominated by alginate and fucoidan that restricts solvent diffusion. Following conventional acidic water extraction (Fig. 4b), the residue remains largely intact, exhibiting only minor surface swelling and limited etching, consistent with the low carbohydrate and protein recoveries obtained. Ultrasound-only treatment (Fig. 4c) produces localised pits, fractures, pores and partial delamination caused by cavitation-induced shear forces and sonoporation, enhancing mass transfer [44], [51]. Further, DES-alone extraction (Fig. 4d) leads to pronounced surface roughening, peeling layers, and widened pores, indicative of chemical disruption of hydrogen-bond networks within carbohydrates and protein-carbohydrate complexes. The most extensive structural collapse is observed in the optimised US-DES residue (Fig. 4e), which exhibits severe fragmentation, high porosity, and loss of ordered structure. This combined mechanical and chemical deconstruction explains the superior carbohydrate and protein recovery achieved under US-DES conditions, as similarly reported for integrated ultrasound-DES extraction systems applied to plant biomasses [52].

**Fig. 4:**
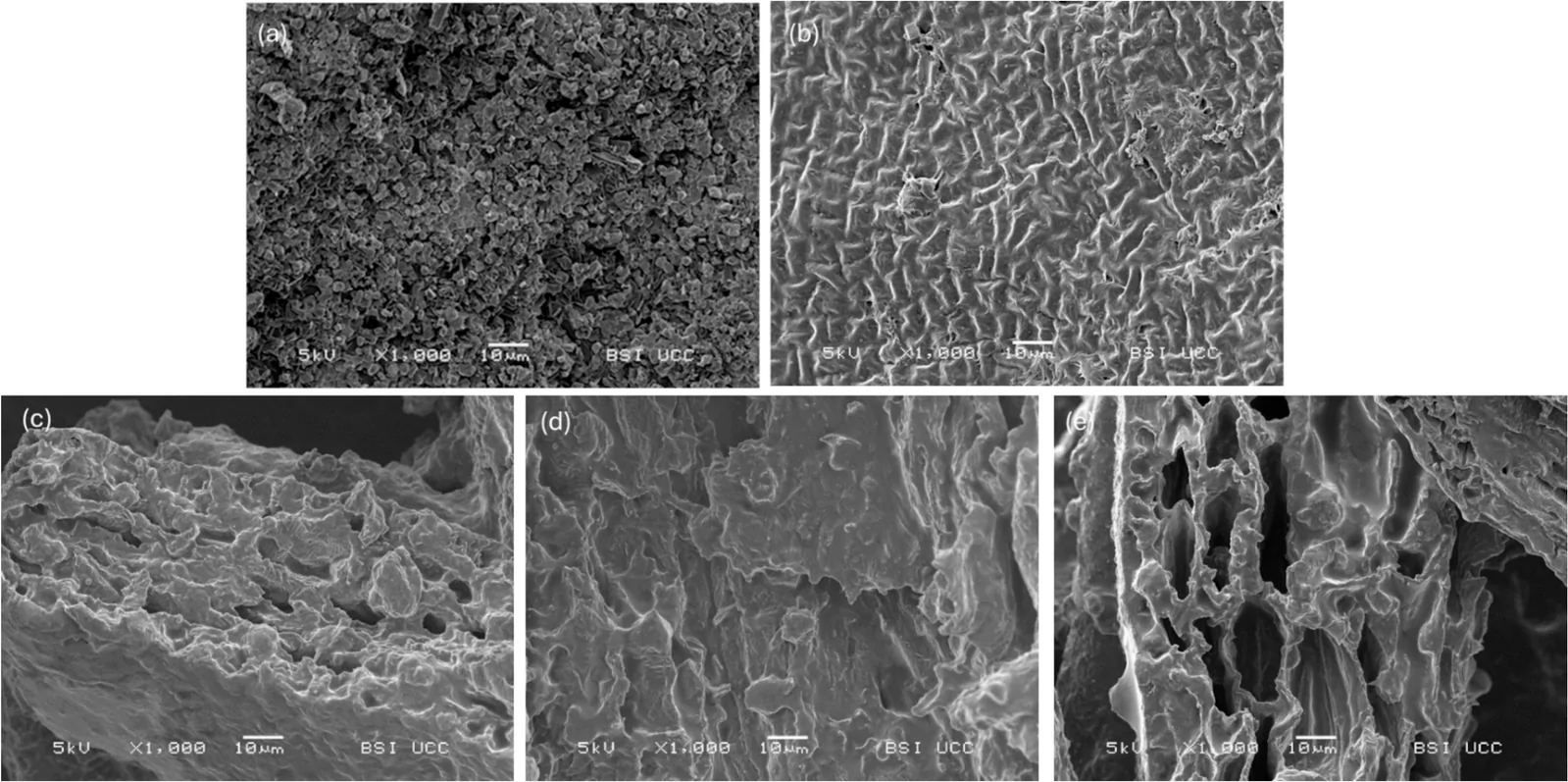
SEM images of (a) ethanol-pretreated, dried, and ground seaweed biomass, (b) residual biomass following conventional extraction, (c) residual biomass following ultrasound-alone extraction, (d) residual biomass following DES-alone extraction, (e) residual biomass following optimised US-DES extraction.

## 4 Conclusions

This study demonstrated the potential of ultrasound-assisted deep eutectic solvent (US-DES) extraction for the simultaneous extraction of proteins and carbohydrates from *Saccharina latissima*. Response surface methodology identified 88% ultrasound amplitude, 12 min sonication time, and 3% solid-to-solvent ratio as the optimised US-DES extraction conditions. Under these conditions, the validated total protein content (TPC) was 50.98 ± 1.29 mg BSAE/g of ethanol-pretreated and dried seaweed, and total soluble carbohydrates (TSC) reached 431.09 ± 17.48 mg GE/g of ethanol-pretreated and dried seaweed. The optimised extract exhibited 73.46 ± 1.30% DPPH radical-scavenging activity and 134.48 ± 1.67 µM TE/mL extract FRAP capacity. Fucose content reached 20.56 ± 0.46 mg FE/g of ethanol-pretreated and dried seaweed, indicating increased recovery of fucose-containing material rather than direct fucoidan quantification. Overall, US-DES provided high extraction performance under mild-temperature conditions. These findings will guide future work focusing on the purification and fractionation of protein-rich and carbohydrate-rich extracts obtained from the DES system, followed by their identification and quantification using high-performance liquid chromatography, which are critical to unlock their full potential. Biological activities and molecular characterisation, including molecular weight distribution and structural elucidation of purified bioactive compounds, are crucial to be performed to ensure extract safety and suitability for downstream applications. These evaluations are essential to support subsequent in vitro and in vivo biological activity studies, ultimately enabling the translation of these extracts into high-value functional food, nutraceutical and biomedical applications.

## 5 Data availability

All data generated and analysed during this study are included within the manuscript.

## CRediT authorship contribution statement

**Rifna E. Jerome:** Writing – review & editing, Writing – original draft, Validation, Software, Methodology, Investigation, Formal analysis, Data curation, Conceptualization. **Nilushni Sivapragasam:** Writing – review & editing, Conceptualization. **Brijesh K. Tiwari:** Writing – review & editing, Resources. **Gaurav Rajauria:** Writing – review & editing, Supervision, Resources, Project administration, Funding acquisition, Conceptualization.

## Declaration of competing interest

The authors declare that they have no known competing financial interests or personal relationships that could have appeared to influence the work reported in this paper.

## Appendix A Supplementary data

Supplementary Data 1 Supporting figures showing the correlation between experimental and model-predicted values for total soluble carbohydrates and total protein content obtained using the response surface methodology (RSM) model, along with standard curves for bovine serum albumin (BSA), D-glucose, Trolox, and fucose used for the estimation of protein content, total soluble carbohydrates, ferric reducing antioxidant power (FRAP), and fucose-rich polysaccharides, respectively.

## References

[1] Garcia-Vaquero M., Ummat V., Tiwari B., Rajauria G. (2020). Exploring ultrasound, microwave and ultrasound–microwave assisted extraction technologies to increase the extraction of bioactive compounds and antioxidants from brown macroalgae. Mar. Drugs.

[2] AOAC, Official Methods of Analysis of AOAC international (16th ed.). Gaithersburg, Maryland, USA., (1996).

[3] Suhre F.B., Corrao P.A., Glover A., Malanoski A.J. (1982). Comparison of three methods for determination of crude protein in meat: collaborative study. J. Assoc. Off. Anal. Chem..

[4] Olsson J., Toth G.B., Albers E. (2020). Biochemical composition of red, green and brown seaweeds on the Swedish west coast. J. Appl. Phycol..

[5] DuBois M., Gilles K.A., Hamilton J.K., Rebers P.A., Smith F. (1956). Colorimetric method for determination of sugars and related substances. Anal. Chem..

[6] Krause M., Sørensen J.C., Petersen I.L., Duque-Estrada P., Cappello C., Tlais A.Z.A., Di Cagno R., Ispiryan L., Sahin A.W., Arendt E.K. (2023). Associating compositional, nutritional and techno-functional characteristics of Faba bean (*Vicia faba* L.) protein isolates and their production side-streams with potential food applications. Foods.

[7] Hewage A., Olatunde O.O., Nimalaratne C., House J.D., Aluko R.E., Bandara N. (2024). Improved protein extraction technology using deep eutectic solvent system for producing high purity fava bean protein isolates at mild conditions. Food Hydrocoll..

[8] Smith P.E., Krohn R.I., Hermanson G., Mallia A., Gartner F., Provenzano M., Fujimoto E., Goeke N., Olson B., Klenk D. (1985). Measurement of protein using bicinchoninic acid. Anal. Biochem..

[9] Pokhrel P., Jha S., Giri B. (2020). Selection of appropriate protein assay method for a paper microfluidics platform. Pract. Lab. Med..

[10] Chen W., Gao L., Song L., Sommerfeld M., Hu Q. (2023). An improved phenol-sulfuric acid method for the quantitative measurement of total carbohydrates in algal biomass. Algal Res..

[11] Y. Olsen, D. Surilayani, A. Handå, Carbohydrate of the brown seaweed, Saccharina Latissima: a review. In: 2nd and 3rd International Conference on Food Security Innovation (ICFSI 2018-2019), Atlantis Press, 2021, pp. 180-182.

[12] Sæther M., Diehl N., Monteiro C., Li H., Niedzwiedz S., Burgunter-Delamare B., Scheschonk L., Bischof K., Forbord S. (2024). The sugar kelp Saccharina latissima II: recent advances in farming and applications. J. Appl. Phycol..

[13] Moldes D., Requejo P.F., Vega M., Bolado S. (2025). Deep eutectic solvent-based protein recovery from microalgal biomass grown in piggery wastewater. Bioresour. Technol..

[14] Benzie I.F., Strain J.J. (1996). The ferric reducing ability of plasma (FRAP) as a measure of “antioxidant power”: the FRAP assay. Anal. Biochem..

[15] Brand-Williams W., Cuvelier M.-E., Berset C. (1995). Use of a free radical method to evaluate antioxidant activity. LWT-Food Sci. Technol..

[16] Usov A., Smirnova G., Klochkova N. (2001). Algae polysaccharides. 55. Polysaccharide composition of some brown Kamchatka algae. Bioorg. Khim.

[17] Lakshmi C., Raju B.D.P., Madhavi T., Sushma N.J. (2014). Identification of bioactive compounds by FTIR analysis and in vitro antioxidant activity of Clitoria ternatea leaf and flower extracts. Indo American J. Pharmacy and Res..

[18] Chemat F., Rombaut N., Sicaire A.-G., Meullemiestre A., Fabiano-Tixier A.-S., Abert-Vian M. (2017). Ultrasound assisted extraction of food and natural products. Mechanisms, techniques, combinations, protocols and applications. A review. Ultrason. Sonochem..

[19] Rifna E., Misra N., Dwivedi M. (2023). Recent advances in extraction technologies for recovery of bioactive compounds derived from fruit and vegetable waste peels: a review. Crit. Rev. Food Sci. Nutr..

[20] S. Kraan, Algal polysaccharides, novel applications and outlook, in: Carbohydrates-comprehensive studies on glycobiology and glycotechnology, IntechOpen, 2012.

[21] Souto-Prieto A., Martinez-Sanz M., Ferreiro T., Parada-Pena P., Abuin-Arias L., Cobos A., Lopez-Sanchez P. (2024). Insights into the structuring ability of two brown seaweeds (*Laminaria digitata* and Saccharina latissima) for applications as natural texturisers. Algal Res..

[22] Kadam S.U., O’Donnell C.P., Rai D.K., Hossain M.B., Burgess C.M., Walsh D., Tiwari B.K. (2015). Laminarin from Irish brown seaweeds Ascophyllum nodosum and Laminaria hyperborea: Ultrasound assisted extraction, characterization and bioactivity. Mar. Drugs.

[23] Kalhor P., Ghandi K. (2019). Deep eutectic solvents for pretreatment, extraction, and catalysis of biomass and food waste. Molecules.

[24] Kundu D., Rao P.S., Banerjee T. (2020). First-principles prediction of Kamlet–Taft solvatochromic parameters of deep eutectic solvent using the COSMO-RS model. Ind. Eng. Chem. Res..

[25] Lu B., Hu H., Yang G., Pan M., Wang Y., Zhang J., Zhang J., Zhang J. (2024). Ultrasound-assisted green and efficient extraction of polysaccharides from Chlorella vulgaris using deep eutectic solvents and improving their performance. New J. Chem..

[26] Shu P., Wang N., Meng Y., Fan S., Liu S., Fan X., Hu D., Yin H., Wang H., Wang X. (2025). Ultrasound-assisted deep eutectic solvent extraction of polysaccharides from Cercis chinensis bark: optimization, kinetics and antioxidant activities. Ultrason. Sonochem..

[27] Li X., Wang J., Huang G., Jia Z., Xu M., Chen W. (2025). Ultrasonic-assisted extraction of polysaccharides from schizochytrium limacinum meal using eutectic solvents: structural characterization and antioxidant activity. Foods.

[28] Jesus M., Romaní A., Santos J., Pires P., Del-Río P., Mata F., Fernandes É., Ramos C., Vaz-Velho M. (2025). High-value brown algae extracts using deep eutectic solvents and microwave-assisted extraction. Foods.

[29] Ling J.K.U., Hadinoto K. (2022). Deep eutectic solvent as green solvent in extraction of biological macromolecules: a review. Int. J. Mol. Sci..

[30] Moldes D., Requejo P.F., Vega M., Bolado S., Wijffels R.H., Kazbar A. (2024). Protein extraction from seaweed Saccharina latissima with deep eutectic solvents. Microchem. J..

[31] Garcia-Vaquero M., O’Doherty J.V., Tiwari B.K., Sweeney T., Rajauria G. (2019). Enhancing the extraction of polysaccharides and antioxidants from macroalgae using sequential hydrothermal-assisted extraction followed by ultrasound and thermal technologies. Mar. Drugs.

[32] Ogutu F.O., Mu T.-H., Elahi R., Zhang M., Sun H.-N. (2015). Ultrasonic modification of selected polysaccharides-review. J. Food Process. Technol..

[33] Zhang J., Ye Z., Liu G., Liang L., Wen C., Liu X., Li Y., Ji T., Liu D., Ren J. (2022). Subcritical water enhanced with deep eutectic solvent for extracting polysaccharides from *Lentinus edodes* and their antioxidant activities. Molecules.

[34] Morais E.S., Lopes A.M.D.C., Freire M.G., Freire C.S., Coutinho J.A., Silvestre A.J. (2020). Use of ionic liquids and deep eutectic solvents in polysaccharides dissolution and extraction processes towards sustainable biomass valorization. Molecules.

[35] Gu J., Lin L., Zhao M. (2023). Demonstration of feasibility and effectiveness of deep eutectic solvent-water system extraction of RG-I type pectin from wolfberry based on target polysaccharide, solvent and their interactions. Food Hydrocoll..

[36] Ummat V., Garcia-Vaquero M., Poojary M.M., Lund M.N., O’Donnell C., Zhang Z., Tiwari B.K. (2021). Green extraction of proteins, umami and other free amino acids from brown macroalgae Ascophyllum nodosum and Fucus vesiculosus. J. Appl. Phycol..

[37] Zin M.T., Kaewkod T., Chaipoot S., Kanthakat G., Chen Y.-Y., Cheirsilp B., Srinuanpan S. (2025). Ultrasound-assisted deep eutectic solvent extraction of antioxidant and anti-colorectal cancer proteins from spirulina biomass: process intensification, characterization, and bioactivity evaluation. Antioxidants.

[38] Vilg J.V., Undeland I. (2017). pH-driven solubilization and isoelectric precipitation of proteins from the brown seaweed Saccharina latissima—effects of osmotic shock, water volume and temperature. J. Appl. Phycol..

[39] Trigo J.P., Stedt K., Schmidt A.E., Kollander B., Edlund U., Nylund G., Pavia H., Abdollahi M., Undeland I. (2023). Mild blanching prior to pH-shift processing of Saccharina latissima retains protein extraction yields and amino acid levels of extracts while minimizing iodine content. Food Chem..

[40] Aasen I.M., Sandbakken I.S., Toldnes B., Roleda M.Y., Slizyte R. (2022). Enrichment of the protein content of the macroalgae Saccharina latissima and Palmaria palmata. Algal Res..

[41] Harrysson H., Hayes M., Eimer F., Carlsson N.-G., Toth G.B., Undeland I. (2018). Production of protein extracts from swedish red, green, and brown seaweeds, Porphyra umbilicalis Kützing, Ulva lactuca Linnaeus, and Saccharina latissima (Linnaeus) JV Lamouroux using three different methods. J. Appl. Phycol..

[42] J. Fleurence, Seaweeds as food, Seaweed in health and disease prevention, (2016) 149-167.

[43] Ummat V., Zhao M., Sivagnanam S.P., Karuppusamy S., Lyons H., Fitzpatrick S., Noore S., Rai D.K., Gómez-Mascaraque L.G., O’Donnell C. (2024). Ultrasound-assisted extraction of alginate from Fucus vesiculosus seaweed by-product post-fucoidan extraction. Mar. Drugs.

[44] Das R.S., Tiwari B.K., Selli S., Kelebek H., Garcia-Vaquero M. (2025). Exploring pilot scale ultrasound-microwave assisted extraction of organic acids and phytochemicals from brown seaweed Alaria esculenta. Algal Res..

[45] Zhang L., Wang M. (2017). Optimization of deep eutectic solvent-based ultrasound-assisted extraction of polysaccharides from Dioscorea opposita Thunb. Int. J. Biol. Macromol..

[46] Obluchinskaya E.D., Pozharitskaya O.N., Lapina I.M., Kulminskaya A.A., Zhurishkina E.V., Shikov A.N. (2025). Comparative evaluation of dynamic maceration and ultrasonic assisted extraction of fucoidan from four arctic brown algae on its antioxidant and anticancer properties. Mar. Drugs.

[47] Olfat A., Mostaghim T., Shahriari S., Salehifar M. (2024). Extraction of bioactive compounds of Hypnea flagelliformis by ultrasound-assisted extraction coupled with natural deep eutectic solvent and enzyme inhibitory activity. Algal Res..

[48] Hu W., Liu B., Wen Y. (2025). Optimization of ultrasound‐assisted deep eutectic solvent extraction and bioactivities of polysaccharide from Porphyra haitanensis. Starch-Stärke.

[49] Zeb L., Gerhardt A.S., Johannesen B.A., Underhaug J., Jordheim M. (2024). Ultrasonic-assisted water-rich natural deep eutectic solvents for sustainable polyphenol extraction from seaweed: a case study on cultivated Saccharina latissima. ACS Sustain. Chem. Eng..

[50] Dai Y., Witkamp G.-J., Verpoorte R., Choi Y.H. (2015). Tailoring properties of natural deep eutectic solvents with water to facilitate their applications. Food Chem..

[51] Rifna E., Dwivedi M. (2022). Effect of pulsed ultrasound assisted extraction and aqueous acetone mixture on total hydrolysable tannins from pomegranate peel. Food Biosci..

[52] Sharma D., Sharma V., Tsai M.-L., Yadav A., Nargotra P., Sun P.-P., Chen C.-W., Dong C.-D. (2025). Improved xylooligosaccharides production from xylan extracted using ultrasound-assisted alcoholic deep eutectic solvent pretreatment of pineapple leaf waste. Ind. Crop Prod..

