# Supplementary material for: Optimisation of ultrasound-assisted deep eutectic solvent extraction of carbohydrates and proteins from farmed brown seaweed (Saccharina latissima) using response surface methodology
Source: Ultrason Sonochem. 2026 Oct 30;134:108144. doi: 10.1016/j.ultsonch.2026.108144 (PMC13755599; doi:10.1016/j.ultsonch.2026.108144)
Supplement: Supplementary file 1 [file mmc1.docx]

Figure S1. Correlation between experimental (actual) and model-predicted values for total soluble carbohydrate (TSC) obtained from the RSM model

Figure S2. Correlation between experimental (actual) and model-predicted values for total protein content (TPC) obtained from the RSM model

Figure S3: Standard curves of bovine serum albumin (BSA) for estimation of protein content

Figure S4. Standard curve of D-glucose for estimation of total soluble carbohydrates (TSC)

| Trolox concentration (µM) | Absorbance |
| --- | --- |
| 10 | 0.0576467 |
| 30 | 0.14798 |
| 50 | 0.246813 |
| 70 | 0.357313 |
| 100 | 0.533813 |
| 120 | 0.69578 |
| 140 | 0.86908 |
| 160 | 0.98778 |

Figure S5. Standard curves of trolox for ferric reducing antioxidant power (FRAP)

| Fucose standard concentration(mg/mL ) | Absorbance |
| --- | --- |
| 0.1 | 1.78823 |
| 0.0625 | 1.13727 |
| 0.0425532 | 0.766267 |
| 0.0217391 | 0.4434 |
| 0.010989 | 0.23555 |
| 0.00552486 | 0.106233 |

Figure S6. Standard curve of fucose for estimation of fucose-rich carbohydrates
